# The 6^th ^Meeting of the Global Alliance to Eliminate Lymphatic Filariasis: A half-time review of lymphatic filariasis elimination and its integration with the control of other neglected tropical diseases

**DOI:** 10.1186/1756-3305-3-100

**Published:** 2010-10-20

**Authors:** David Addiss, The Global Alliance to Eliminate Lymphatic Filariasis

**Affiliations:** 1 Holos Associates, 1626 Grove St, Kalamazoo, MI 49006 USA; 2 Secratariat of the Global Alliance to Eliminate Lymphatic Filariasis, Centre for Neglected Tropical Diseases, Liverpool School of Tropical Medicine, Pembroke Place, Liverpool L3 5QA, UK

## Abstract

The 6^th ^Meeting of the Global Alliance to Eliminate Lymphatic Filariasis (GAELF6) was held 1-3 June, 2010 in Seoul, Korea, with 150 participants from 38 countries. The year 2010 marks the midpoint between the first GAELF meeting, in 2000, and the World Health Organization (WHO) 2020 goal of global elimination of lymphatic filariasis (LF) as a public health problem. The theme of the meeting, "Half-time in LF Elimination: Teaming Up with Neglected Tropical Diseases (NTDs)," reflected significant integration of LF elimination programmes into a comprehensive initiative to control NTDs. Presentations on LF epidemiology, treatment, research, and programmes highlighted both accomplishments and remaining challenges.

The WHO strategy to interrupt LF transmission is based on annual mass drug administration (MDA) using two-drug combinations. After mapping the geographic distribution of LF, MDA is implemented for ≥ 5 years, followed by a period of post-MDA surveillance, and, ultimately, verification of LF elimination. Morbidity management further reduces disease burden.

Of 81 countries considered LF-endemic in 2000, 52 (64.2%) have begun MDA; 10 (12.3%) others with low-level transmission are unlikely to require MDA. In 2008, ~695 million people were offered treatment (51.7% of the at-risk population); ~496 million participated. Approximately 22 million people have been protected from LF infection and disease, with savings of ~US $24.2 billion. Morbidity management programmes have been implemented in 27 (33.3%) countries.

Significant challenges to LF elimination remain. These include: initiating MDA in the remaining 19 countries that require it; achieving full geographic coverage in countries where MDA has started; finding alternative strategies to address the problem of *Loa loa *co-endemicity in Central Africa; developing strategies to treat urban populations; initiating and sustaining MDA in settings of armed conflict; developing refined guidelines and procedures for stopping MDA, for post-MDA surveillance, and for verifying the elimination of LF; and integrating morbidity management into all LF elimination programmes. Scientific research and enhanced advocacy for NTDs remain critical for addressing these challenges.

GAELF6 was characterized by enthusiasm and recognition that "teaming up with NTDs" offers opportunities for new partnerships, fresh perspectives, enhanced advocacy, and greater programmatic integration in a rapidly changing global health environment.

## Executive Summary

The 6^th ^Meeting of the Global Alliance to Eliminate Lymphatic Filariasis (GAELF) was held 1-3 June, 2010 in Seoul, Korea. The theme of the meeting, "Half-time in LF Elimination: Teaming Up with NTDs," highlighted the fact that 2010 marks the midpoint between the first GAELF meeting, held in 2000 in Santiago de Compostela, Spain, and the 2020 World Health Organization (WHO) goal of global elimination of lymphatic filariasis (LF) as a public health problem. The "half-time" theme provided an appropriate backdrop for reflection, assessment, and planning. For many participants, it also coincided with eager anticipation of the World Cup, which began in South Africa three weeks later.

Given the half-time theme, it was especially fitting that GAELF6 was held in the Republic of Korea, which in 2008 received official WHO verification that it had reached its goal of LF elimination. GAELF participants appreciated the opportunity to learn about the successful effort in Korea. The warm hospitality and seamless organization of the Korean hosts provided a superb and relevant setting for this important meeting.

The presentations, by global experts on the epidemiology, treatment, research, and programme implementation of LF, highlighted the impressive accomplishments of the GAELF's first 10 years and provided insight into the major remaining challenges facing the GAELF. Both accomplishments and challenges were articulated by several "case studies" from LF-endemic countries. Formal and informal discussions were lively and focused. Two themes, in particular, infused almost every presentation and discussion. First, LF elimination is increasingly integrated into a larger initiative to control neglected tropical diseases (NTDs); hence, the theme "Teaming up with NTDs." Implications of this "teaming up" will affect all aspects of the GPELF (Global Programme to Eliminate Lymphatic Filariasis), including drug regimens, advocacy, governance, financing, monitoring and evaluation, partnerships, morbidity management, vector control, research, and programme implementation. Secondly, the global health landscape has undergone remarkable changes since the GAELF was established, and this has resulted in new partnerships, initiatives, and funding opportunities. These changes will only accelerate during the next 10 years and foresight, flexibility, and strength of purpose will all be required.

### Half-time Assessment

The magnitude of the challenge facing the GAELF in 2000 was staggering. 81 countries were considered endemic for LF, with 1.34 billion persons at risk of infection and 120 million infected. WHO, as the lead agency in the GPELF, established two major strategies to achieve the 2020 goal: 1) stopping the spread of infection through annual mass drug administration (MDA) using two-drug combinations; and 2) reducing the burden of disease through morbidity management. WHO recommended a stepwise approach to interrupt LF transmission, beginning with mapping the distribution of LF to identify areas in need of MDA, followed by five or more years of MDA, a period of post-MDA surveillance, and, ultimately, verification of LF elimination.

Despite the challenges, progress toward LF elimination has been impressive. Of the 81 endemic countries, 10 (12.3%) are unlikely to require MDA based on current assessment and 52 (64.2%) currently have active MDA programmes. Of these, 37 countries have completed ≥ 5 rounds of MDA in at least some of their endemic areas. In 2008, an estimated 496 million people participated in MDA; 695 million were offered treatment, representing 51.7% of the at-risk population. That same year, the cumulative number of albendazole tablets donated by GlaxoSmithKline (GSK) for LF elimination reached 1.4 billion and the number of Mectizan^® ^tablets donated by Merck & Co., Inc. reached 1.2 billion.

The resulting global impact on health has been enormous. An estimated 22 million people have been protected from LF infection and disease, with estimated economic savings of US $24.2 billion. Declines in microfilaremia prevalence have been reported from 131 sentinel sites after 5 rounds of MDA; 68 (63%) had a 100% reduction in prevalence. Morbidity management programmes implemented in 27 (33.3%) of the 81 LF-endemic countries have shown significant reductions in acute inflammatory episodes in persons with lymphoedema. An estimated 146 million persons are estimated to have received "beyond-LF" benefits during the first 8 years of the programme, due to the broad anti-parasitic activity of the donated drugs.

### Challenges for the Next Half

Several significant challenges remain if LF is to be globally eliminated as a public health problem by 2020. Various aspects of these challenges were highlighted by many of the GAELF6 speakers, revealing a strong sense of consensus on what the challenges are and what may be needed to meet them. Common themes as to "what is needed" include even greater advocacy and funding; carefully targeted operational research, which can readily be applied in the field; deepened and expanded partnerships; persistence; and flexible approaches. Again, all of this will be taking place within an integrated NTD context and a rapidly changing global health environment. Key challenges include the following:

#### Getting started

Nineteen countries that require MDA have not yet begun. To reach the global goal of 2020, initiating MDA in these countries, 16 of which are in Africa, is a priority.

#### Upscaling

In the countries where MDA has already begun, it is critical to upscale MDA to full geographic coverage. 70% of the total at-risk target population, 919.5 million people, live in the countries of India, Indonesia, Bangladesh, Nigeria, and the Democratic Republic of Congo. Full geographic coverage, which has been achieved by India, is a priority for the other four countries.

#### *Loa loa*

In Central Africa, *Loa loa *co-endemicity has presented a major barrier to initiating LF elimination programmes. Research is underway, and results are urgently needed, to find and test alternative or provisional strategies.

#### Urban populations

Strategies must be developed to effectively treat urban populations where this is needed, particularly in Africa and Asia.

#### Conflict and post-conflict settings

Of the 19 countries with active LF transmission that have not yet begun MDA, 13 have fragile infrastructures or are in post-conflict situations. Experience has shown that MDA is possible in such settings, if special precautions and principles are adhered to.

#### Post-MDA surveillance

An urgent need exists for refined guidelines for stopping MDA and for post-MDA surveillance.

#### Verification of elimination

Procedures, guidelines, and criteria for verifying the elimination of LF are needed so that formerly-endemic countries can be "taken off the list" as they reach their goal.

#### Morbidity management and disability prevention

Only 27 LF-endemic countries have active morbidity management and disability prevention programmes. Morbidity management should be part of all LF elimination programmes. Integrated NTD case management offers the promise of new partnerships and broader integration of LF morbidity management into existing health services.

### Opportunities and Resources

Speakers at GAELF6 highlighted the lessons that have been learned during the first 10 years of the partnership. These lessons provide insights, as well as opportunities, to address the remaining challenges.

#### GAELF

The open, inclusive nature of the GAELF, with its "light" governance structure and regional approach, provides a solid foundation for meeting the 2020 goal and for leadership within an integrated NTD initiative.

#### Human resources and goodwill

In addition to the GAELF, the most important resource for success lies in the strength and dedication of the many thousands of people involved in LF elimination across the globe. As Dr Mwele Malecela said in one of her presentations, "It is your commitment, your passion, your belief in the possibility of LF elimination that gives the Alliance its strength."

#### Research

Participants at the GAELF6 learned of several major research initiatives to address obstacles to LF elimination. Studies are underway on the impact of vector control on LF transmission; alternative drug regimens and drug dosing; new macrofilaricidal agents; and assessment of diagnostic tools, including xenomonitoring of vectors to detect filarial DNA, among others.

#### Funding and support

In the last few years, significant new funding has been committed to LF by the UK Department for International Development (DFID), the US Agency for International Development (USAID), and the Bill & Melinda Gates Foundation. The commitment by GSK and Merck & Co., Inc. to donate drugs as well as provide other support remains essential and strong. GAELF6 participants learned of line-item funding for LF elimination in Ministry of Health budgets and of the fund established by the President of Tanzania for hydrocele surgery. Thanks in part to the integration of LF with other NTDs, advocacy for funding is more successful than ever before as in many countries the LF programme establishes the critical platform.

#### Integration

As noted above, "teaming up with NTDs" offers many opportunities for new partnerships, fresh perspectives, enhanced advocacy, and a greater role for programmatic aspects that have to date received limited attention (e.g., vector control and morbidity management).

### Conclusion

The GAELF6 meeting provided a rich opportunity for 150 attendees from 38 countries to take stock, reflect, celebrate, and plan for the future. A sense of enthusiasm and celebration infused the meeting, as well as recognition and anticipation of the challenges ahead. A strong sense of partnership was palpable, which bodes well for the next 10 years.

## Tuesday, 1 June 2020

### Opening Session

**Chair**: Dr Jong-Koo Lee Deputy Minister/Director, Ministry of Health and Welfare, Korea Centers for Disease Control and Prevention (KCDC)

Dr Young-Hak Yoo, Honourable Deputy Minister of Health and Welfare of Korea, opened the 6^th ^meeting of the Global Alliance to Eliminate Lymphatic Filariasis (GAELF). He welcomed the 150 participants from 38 countries and expressed appreciation for the opportunity to share Korea's successful experience with elimination of lymphatic filariasis (LF). He voiced hope that the meeting would strengthen international cooperation in the global effort to eliminate LF.

Dato Dr Tee Ah Sian, Director of the Division of Combating Communicable Diseases, Western Pacific Region (WPR) of the World Health Organization (WHO), thanked the government of Korea for hosting the meeting and welcomed the participants. She highlighted the substantial physical and psychological burdens of LF and noted progress in developing effective laboratory tools and public-private partnerships to eliminate LF. Several countries in the WPR appear to be close to eliminating LF and others have made substantial progress. Dr Tee called upon the GAELF to assist Papua New Guinea in mobilizing the financial and technical resources necessary to eliminate LF.

Dr Dirk Engels, Coordinator of Preventive Chemotherapy & Transmission Control (PCT) at WHO in Geneva, conveyed greetings from Dr Margaret Chan, WHO Director-General, and Dr Lorenzo Savioli, Director of the Department of Control of Neglected Tropical Diseases. He remarked that, in 2008, the Global Programme to Eliminate Lymphatic Filariasis (GPELF) targeted 695 million people with mass drug administration (MDA) and treated 496 million [1]. The strength of the GAELF lies in its members, each with a different mandate, but all sharing the same goal. Dr Engels reviewed some of GAELF's successes and reiterated WHO's full support. He emphasized that MDA for LF elimination should be thoroughly integrated with preventive chemotherapy for neglected tropical diseases (NTDs). He thanked GlaxoSmithKline (GSK) and Merck & Co., Inc. for their continued commitment to provide antifilarial drugs, free of charge, for as long as necessary. Dr Engels encouraged the meeting participants to celebrate the successes already achieved and noted that the LF elimination programme already has been successful in improving the health of millions of poor people.

On behalf of the delegates to the 6^th ^GAELF, Professor David Molyneux, Executive Secretary of the Executive Group, GAELF, extended his thanks to the Korean hosts of the meeting and noted the significance of holding the meeting in a country that has successfully eliminated LF. Professor Molyneux emphasized the dramatic changes in the global health agenda during the first 10 years of the GAELF's history, especially the substantial funds recently pledged to control and eliminate NTDs. He stated that LF elimination is at the forefront of NTD programmes, and that this was made possible by drug donations from GSK and Merck & Co., Inc..

The first 10 years of the GAELF have demonstrated that elimination of LF as a public health problem is an achievable goal, with enormous public health benefits. Professor Molyneux noted that the GPELF, while maintaining its LF focus, can serve as a platform for integration with other NTDs. The features of the GAELF - a loose, non-restrictive, representative governance structure, regional approaches, mutual respect, and the ability to learn lessons and adapt - have made it strong and attractive to a diverse array of partners.

Dr Mwele Malecela, Acting Director-General, National Institute for Medical Research, United Republic of Tanzania thanked the GAELF members, especially the Executive Group and the Secretariat, for their support during her four years as President of GAELF. She reflected on some of the lessons learned during the past four years. These lessons include: partnership is a process; consensus can emerge from chaos; and advocacy can move mountains. The flexible nature of GAELF has allowed its members to forge new partnerships and to move from strength to strength. She thanked the government of the Republic of Korea and the KCDC for hosting the meeting and told the participants that, "it is your commitment, your passion, your belief in the possibility of LF elimination that gives the Alliance its strength."

Dr Jong-Koo Lee, Deputy Minister/Director, Ministry of Health and Welfare, KCDC, thanked the GAELF for the opportunity to host GAELF6. He noted that, before LF elimination was verified by WHO in 2008, LF had been endemic in Korea for millennia. LF was the first disease to be officially eliminated in Korea. Dr Lee pledged that during the next decade, global health will remain high on Korea's development agenda. He wished the participants much success and declared the meeting officially opened.

### Keynote Addresses

**Chair**: Professor Jong-Yil Chai, Seoul National University College of Medicine

The first session of the conference, chaired by Professor Jong-Yil Chai, described the successful elimination of LF in Korea. Professor Han-Jong Rim, Emeritus Professor at Korea University College of Medicine, reviewed the history of LF elimination in Korea, which can be considered in three distinct phases [2]. During the first phase, 1920-1945, LF was initially recognized as an endemic public health problem and was found to be caused only by *Brugia malayi*.

The period 1951-1979 was one of investigation and surveillance. Three major endemic areas were identified: Jeju Island; the southwest coastal area; and an area in the southeast, which extended inland. Epidemiologic investigations were conducted in all of these areas, where microfilaremia prevalence ranged from 1 to 22%. These investigations showed that *B. malayi *was nocturnally periodic and the principal vectors were *Aedes togoi *in the coastal areas, and *Anopheles sinensis *inland. No LF transmission was detected in the central part of the country, which is mountainous. Persons infected with *B. malayi *were treated selectively with diethylcarbamazine (DEC), beginning with low doses to minimize adverse reactions. Certain aspects of traditional life were identified as facilitating transmission, including the habit of gathering water during the evenings, and the thatched roofs, which provided resting places for *A. togoi*.

**Figure 1:**
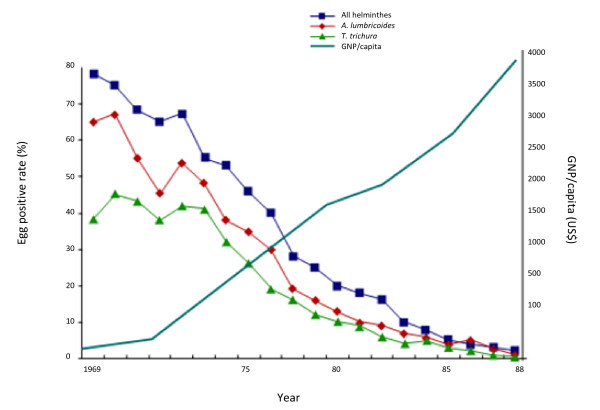
**Prevalence of infection with soil-transmitted helminths and per-capita gross national product (GNP), Republic of Korea, 1969-1988**.

The third phase, from 1980 to 2007, focused on chemotherapy and control of LF. Dr Rim noted that Korea did not have a centralized national LF control programme. Rather, the Korean National Institute of Health conducted and coordinated LF control efforts that were carried out by a variety of investigators. The primary control measures included epidemiologic investigation and surveillance along with selective chemotherapy, primarily with DEC. Economic growth and improved living conditions, which facilitated lifestyle changes, decreased exposure to mosquitoes. A progressive socio-economic development plan, known as "Saemaul Undong," was launched in 1970. This public works programme improved housing conditions, did away with traditional thatched roofs, and decreased mosquito breeding habitat; it also dramatically increased per-capita income. The prevalence of *B. malayi *infection declined rapidly, as did the prevalence of soil-transmitted helminths (STHs), from 80% in 1969 to nearly zero in 1989 (Figure 1). In commenting on the relationship between economic development and parasitic diseases, Dr Rim said that social and environmental change, made possible by economic growth, was primarily responsible for the dramatic decline in soil-transmitted diseases and LF in Korea.

Dr Hyeng-Il Cheun, Research Fellow, Division of Malaria and Parasitic Diseases, National Institute of Health, KCDC, gave additional detail on the LF elimination programme in Korea, focusing on epidemiological surveys and elimination efforts between 2002 and 2006 [3]. The objectives during this period were to enhance surveillance in suspected areas; detect infected persons; provide selective and mass chemotherapy; confirm the absence of infection in vector mosquitoes; and verify LF elimination. Surveys were repeated on many of the islands, and previous efforts to reduce transmission were found to have been effective. For example, in Jeollanam-do, microfilaremia prevalence was 9.8% in 1986-1992. In 2002, only two persons, both > 60 years old, were found to be infected, for a prevalence of 0.1%. In other areas where LF prevalence had been lower in 1992, no one tested positive in 2002-2004.

In October 2005, a team from WHO visited Korea and recommended that 3000 elementary school children and older residents of formerly-endemic areas be tested with a rapid antibody test for *Brugia malayi*. All children had negative test results. In March 2008, WHO officially concluded that the Republic of Korea had achieved the elimination of LF as a public health problem.

#### Address by GlaxoSmithKline

Dr Yil-Seob Lee, Medical and Regulatory Director of GlaxoSmithKline in Korea, gave an overview of GSK's humanitarian efforts. To fulfil its commitment to provide albendazole for LF elimination, GSK opened a new albendazole manufacturing facility in India in 2009. Among its other humanitarian programmes, GSK sponsors research on diseases endemic in the poorest countries; re-invests in the least-developed countries 20% of profits made on medicines sold in those countries; and shares its laboratory space with independent researchers working on neglected diseases. Dr Lee emphasized the importance of partnership and collaboration in achieving the goal of LF elimination.

#### Address by Merck Sharpe & Dohme

Mr Key Lee, head of External Affairs, Merck Sharpe & Dohme (MSD) in Korea, noted both the tremendous progress made in LF elimination thus far and the magnitude of the work ahead. In 1998, the Mectizan Donation Program (MDP) was expanded to target LF elimination in 28 African countries and Yemen, where onchocerciasis was co-endemic. Last year, > 100 million doses of Mectizan^® ^were approved for combination therapy against LF. Dr Lee reiterated MSD's commitment to provide Mectizan^® ^free of charge, for as long as needed, both for onchocerciasis control and LF elimination. He urged the GAELF participants not to forget those who suffer from filarial disease. Dr Lee called for continued momentum through strengthened partnerships to address NTDs, and briefly highlighted MSD's other humanitarian programmes in Korea.

### First Half: The Journey from Santiago de Compostela to Seoul

**Chair**: Dr Mwele Malecela

#### The Alliance Journey: The Changing Environment and Adapting the Game Plan

Professor David Molyneux, Senior Professorial Fellow, Centre for Neglected Tropical Diseases, Liverpool School of Tropical Medicine, reflected on how dramatically the international health environment has changed since the first GAELF meeting in Santiago de Compostela in 2000. Some of these milestones include the release of the Report of the Commission on Macroeconomics and Health, chaired by Dr Jeffrey Sachs; the adoption of the Millennium Development Goals (MDGs); the establishment of the Global Fund for AIDS, Tuberculosis, and Malaria; the emergence of numerous public/private partnerships; funding support from the Bill & Melinda Gates Foundation for international health research; the emergence of the "NTD brand" following seminal meetings in Berlin; and increased references to NTDs by the Director-General of WHO and national leaders in Europe and North America.

Professor Molyneux described "major routes" on the journey of the GAELF. These have included a global commitment to LF elimination; a strong research agenda, with support from numerous public and private institutions; and enhanced advocacy for NTDs.

The GAELF also has travelled extensively on "country roads," which include years of commitment to LF control before 1997 by the governments of China, Korea, India, Thailand, Sri Lanka, and Suriname amongst others; a unified embrace of WHO's strategy for LF; successful efforts to "scale up" programmes to the national level in many countries; and the substantial financial contributions to LF elimination made by filariasis-endemic countries themselves.

Professor Molyneux outlined several "roadblocks" to global LF elimination, including *Loa loa *in West Africa; challenges to upscaling programmes in countries where the greatest LF burden remains; conflict and post-conflict situations in several countries; supply and financing of DEC; and morbidity control. He concluded with the phrase, "Many roads - one journey - same destination: LF elimination."

#### Goals Scored: Progress Achieved in LF Elimination

Dr Kazuyo Ichimori, WHO Focal Point for Lymphatic Filariasis Elimination, reviewed the key milestones in GPELF's 10-year history and provided an assessment of its impact and the major remaining challenges. The data that she presented represented official WHO statistics as of May 2010.

The GPELF had its origins following the World Health Assembly (WHA) resolution 50.29, passed in 1997, which urged member states to eliminate LF as a public health problem. WHO published a strategic plan for LF elimination in September 1999, and the GAELF held its first meeting in 2000 in Santiago de Compostela, Spain. WHO is currently preparing a report that will review progress from 2000 to 2009 and lay out a strategic plan for LF elimination for 2010-2020.

The GPELF set as its goal the global elimination of LF as a public health problem by 2020, to be achieved through two major strategies: 1) stopping the spread of infection by using MDA; and 2) reducing the burden of disease through morbidity management. A stepwise programmatic strategy to interrupt transmission has been recommended, beginning with mapping to assess areas where MDA is needed; followed by five or more years of MDA using a combination of two drugs for every eligible individual in endemic areas. This is followed by a period of post-MDA surveillance; and, ultimately, verification of LF elimination.

**Figure 2:**
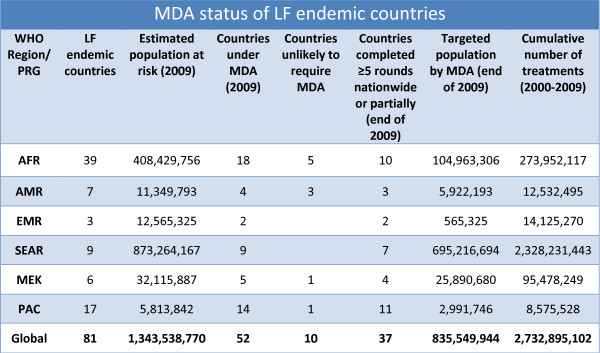
**Progress towards LF elimination and status of mass drug administration (MDA) in 81 LF-endemic countries, by WHO region**.

Currently, 81 countries are considered endemic for LF, with 1.34 billion persons at risk of infection and 120 million infected. Of the total global burden, 65% is found in the Southeast Asia Region of WHO (SEAR), followed by the Africa Region (AFR), with 35%. As shown in Figure 2, of the 81 endemic countries, 10 (12.3%) are unlikely to require MDA based on current assessment and 52 (64.2%) currently have active MDA programmes. Of these, 37 countries have completed ≥ 5 rounds of MDA in at least some of their endemic areas. In the African region, progress has been somewhat slower; of 39 LF-endemic countries in the African region, 18 (46.2%) have active MDA programmes.

In 2008, treatment was offered to 695 million people, representing 51.7% of the at-risk population [1]. That same year, the cumulative number of albendazole tablets donated by GSK for LF elimination reached 1.4 billion, while Merck & Co., Inc. had donated 1.2 billion tablets of Mectizan^® ^[4].

**Figure 3:**
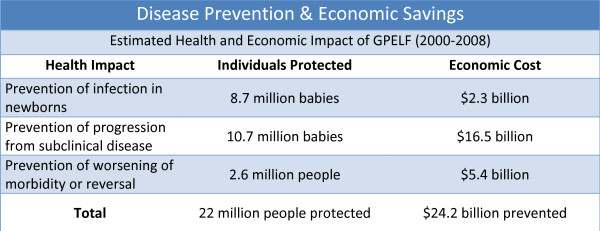
**Estimated health and economic impact of the Global Programme to Eliminate Lymphatic Filariasis (GPELF), 2000-2008**.

The resulting global impact and benefits have been enormous. An estimated 22 million people have been protected from LF infection and disease, with economic savings of US $24.2 billion (Figure 3) [5]. Declines in microfilaremia prevalence have been reported from 131 sentinel sites after 5 rounds of MDA; 68 (63%) had a 100% reduction in prevalence and another 21% had reductions of 75-99%. Two key factors, baseline microfilaremia prevalence and compliance with MDA, influenced the degree of reduction in microfilaremia.

**Figure 4:**
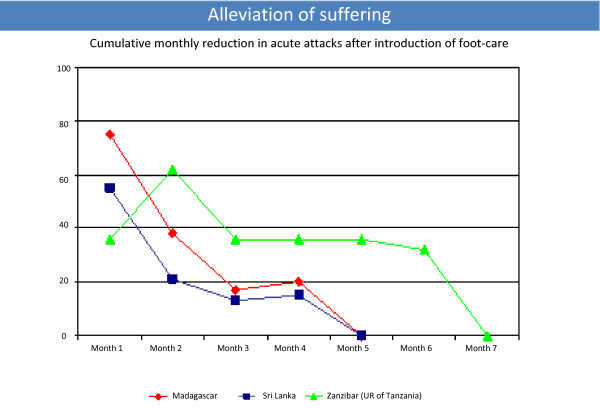
**Cumulative percent reduction in episodes of adenolymphangitis (acute attacks) after introduction of basic lymphoedema management ("foot-care") in three countries**.

Morbidity management programmes have been implemented in 27 (33.3%) of the 81 LF-endemic countries. Assessment of several of these programmes has revealed significant reductions in episodes of adenolymphangitis (ADL), or "acute attacks," in persons with lymphoedema (Figure 4) [6-8]. Auxiliary benefits of the GPELF include reductions in the prevalence and intensity of infection with STHs and decreases in onchocerciasis, scabies, and other ectoparasite infections in areas where these are co-endemic with LF.

Key remaining challenges include: initiating MDA in large urban settings and in the endemic African countries that have not yet started MDA, especially where *Loa loa *is co-endemic; implementing and expanding morbidity management to all LF-endemic countries; developing and testing guidelines for stopping MDA and for implementing post-MDA surveillance; and developing a process to ascertain and verify elimination of LF.

#### Half-time Around the World: Case Studies

##### Elimination of Lymphatic Filariasis in India

Dr PK Srivastava, Joint Director of the National Vector Borne Disease Control Programme, Ministry of Health and Welfare, reported that LF is endemic in 250 districts in 20 states in India, with an at-risk population of 600 million. In 2009, MDA was conducted in all endemic districts with co-administered DEC and albendazole. MDA coverage (the percentage of the eligible population that receives antifilarial drugs) averaged 85%. Compliance (the percentage that actually takes the drug) was lower, but this figure is improving. The overall prevalence of microfilaremia decreased from 1.24% in 2004 to 0.53% in 2008 [9].

Challenges for the India programme include the need for improved social mobilization and supervision to increase compliance with MDA, especially in urban areas; maintaining adequate supply and improving handling and storage of antifilarial drugs; access to technical expertise for monitoring and evaluation of such a massive programme; monitoring and surveillance in implementation units (IUs) that have met current WHO criteria for stopping MDA; and expanding morbidity management activities.

##### LF Elimination in Papua New Guinea

Dr Leo Sora Makita, Health Advisor, Malaria and Vector Borne Disease, National Department of Health, discussed LF elimination in Papua New Guinea, where an estimated 1 million of its 6.2 million inhabitants are infected with *Wuchereria bancrofti *and 3 million are at risk of infection. The prevalence of infection is as high as 92% in East Sepik Province. Although the national health plan, adopted in 2001, called for MDA and morbidity management in LF-endemic areas, progress has been slow due to the substantial challenges of dense forests, rugged terrain and swamps; limited infrastructure; a highly scattered population speaking 823 different languages; insufficient human resources; and lack of sustained financial support. The current plan is to complete LF mapping throughout the country and to implement MDA in two provinces, adding one new province each year.

##### The Road to LF Elimination in the Philippines

Dr Leda Hernandez, Division Chief, Infectious Disease Office, National Center for Disease Prevention and Control, Department of Health, highlighted progress in the Philippines. Of 80 provinces, 43 are considered endemic for LF. MDA has been implemented in 38 provinces, with a mean coverage of 70% (range, 68-89%). In 2010, the plan is to conduct MDA in all IUs where the prevalence of microfilaremia is > 1%. Morbidity management has developed in partnership with non-governmental development organizations (NGDOs) that have interest in hydrocele surgery and home-based disability care. Guidelines on disability prevention have been developed and will be disseminated this year.

Mid-term surveys have documented reductions in the prevalence of microfilaremia and antigenemia in the IUs, reaching the level required for elimination in 6 provinces.

Key factors facilitating success of the programme have included: the prioritizing of diseases for elimination by leading health policy-makers; establishment of a separate budget within the Ministry of Health for LF elimination; partnerships with other governmental sectors and with local and international NGDOs; executive leadership; and interest in integrated delivery of health services.

##### Progress Achieved in LF Elimination in Yemen

Dr Abdul Samid Al-Kubati, National Focal Point for Lymphatic Filariasis, Ministry of Public Health described the successful elimination of LF in Yemen. In 2000-2002, 65 suspected LF-endemic districts were mapped using ICT card tests. Of these, 9 were found to be endemic, with antigenemia prevalences of 2-40%. MDA was conducted between 2002 and 2009 in these districts, with an overall population at risk of ~100,000. By 2006, all IUs completed 5 rounds of MDA; all but one had reached the criteria for stopping MDA. With three more annual rounds of treatment, the criteria for stopping MDA were reached in this last IU. Vector control and morbidity management were part of the programme. Current plans are to conduct passive laboratory-based surveillance as well as biennial surveys for infection in the 6-8 year age group using ICT card tests.

##### LF Elimination in Madagascar

Dr Lisy Rasoazanamiarana, National Coordinator, Lymphatic Filariasis Elimination Programme, Ministry of Public Health, presented highlights of the programme in Madagascar, which has treated 30 of its endemic districts using combined DEC and albendazole. The prevalence of microfilaremia in the sentinel sites has been dramatically reduced. Morbidity management has been implemented in collaboration with WHO and NGDOs such as Reggio Terzo Mundo, Handicap International, and Azafady, and lymphoedema management has been integrated with leprosy care. In the 30 IUs where MDA has been implemented, 8308 cases of lymphoedema and 7710 cases of hydrocele have been registered. In 2009, 752 hydrocele surgeries were performed and 3525 lymphoedema cases were being managed. Challenges include inadequate financing and increasing demands by drug distributors for remuneration.

##### National NTD Control Programme in Haiti

Dr Abdel Nasser Direny, IMA-World Health Country Representative and NTD Programme Manager, described the national NTD control programme in Haiti, which has addressed both LF and STHs. LF elimination started in 2000 with a pilot programme in the town of Leogane. By 2005, the national programme included 24 Communes, but it was temporarily suspended later that year due to lack of funds. The current integrated NTD programme in Haiti is jointly sponsored by the Ministry of Health (MSPP) and the Ministry of Education (MENFP) and is supported financially by the NTD programme of the US Agency for International Development (USAID) and Research Triangle Institute (RTI), as well as by a grant from the Bill & Melinda Gates Foundation to the University of Notre Dame.

In 2009-2010, 2.8 million persons were treated, for an estimated drug coverage of 96% of the total population (based on imprecise population figures). Morbidity management is currently limited to a clinic in Leogane, although some 7000 patients with lymphoedema and 700 with hydrocele have been treated. Dramatic reductions in prevalence of LF and STH infections were observed between 2000 and 2005 in Leogane, although increases in prevalence were seen after MDA was interrupted in 2005 [10].

The massive earthquake on January 12, 2010 temporarily halted the NTD programme, although training and MDA are scheduled to restart this summer. Indeed, Haiti has a goal of achieving national-level geographic coverage in 2011, including the urban area of Port au Prince.

#### Goals Scored: Health Impact Achieved

Dr Eric Ottesen presented the results of an analysis of the health impact of the LF elimination programme during its first 8 years (2000-2007) [4]. The analysis includes health improvements directly related to reduced transmission of LF and those "beyond-LF." The latter benefits, primarily resulting from the broad anti-parasitic effectiveness of albendazole and ivermectin, include decreased intestinal worm infections in children; reduced hookworm-related disease in women of child-bearing age; and relief of debilitating skin diseases. Data for this assessment came from the scientific literature on mechanisms of disease and from existing data on drug efficacy, MDA coverage, and the age and gender distribution of populations in filariasis-endemic countries.

The results suggest that the LF elimination effort is having a huge impact, with some 16.1 million persons receiving direct LF benefits (6.6 million newborns protected and disease progression halted in 9.5 million), and 32 million disability-adjusted life years (DALYs) averted. An estimated 146 million persons are estimated to have received "beyond-LF" benefits during the first 8 years of the programme, including 56.6 million children treated for intestinal worms, 44.5 million women of childbearing age treated for STHs, and 45 million persons receiving relief of debilitating skin diseases. Dr Ottesen noted the many challenges and opportunities that remain to better quantify the health benefits of LF elimination.

#### Goals Scored: Economic Impact

Mr Brian Chu reviewed the economic benefits of the first 8 years of the GPELF. These benefits are experienced by persons with LF infection, who are protected from *progression *of disease; by individuals in affected communities who are protected from *acquiring *infection (and subsequent disease); and by the public health *systems *in filariasis-endemic areas. The analysis is based on the direct and indirect costs averted as a result of preventing hydrocele, lymphoedema and ADL. Based on the available knowledge of these diseases and the health impact analysis presented by Dr Ottesen, the programme's economic benefit to date is conservatively estimated at $24 billion (Figure 3). The analysis suggests that 78% of benefits are related to chronic disease, compared to 22% for acute disease, and that the vast majority of benefits ($20.2 billion) have been achieved in Asia (SEAR) [5].

Mr Chu cautioned that the "beyond-LF" benefits are hard to quantify, and therefore were not included in the model. If 100% of the at-risk population were covered under MDA, the model estimates that economic benefit would reach $55 billion. The largest economic gaps (economic benefits yet to be realized) are in the African region. In conclusion, Mr Chu noted that the GPELF is an excellent investment in global health, with impressive economic rates of return. The success of the GPELF, measured in economic terms, is a strong affirmation of the value of investing global health resources in targeting the NTDs.

#### Goals Scored: In Partnership

Dr Adrian Hopkins, Director of the Mectizan Donation Program, spoke on the crucial role of partnerships in LF elimination. He defined partnership as a *relationship *between individuals or groups of people, characterized by *mutual cooperation and responsibility*, for the *achievement of a specified goal*. Partnerships develop and evolve fruitfully if several conditions are met, including commitment to common goals; clarity on the role of each partner; and having established ways to resolve conflict - which is inevitable.

Dr Hopkins noted that the GAELF involves a "remarkable mix of partners," bringing together Ministries of Health and Finance with various multilateral and bilateral donors and NGDOs. Corporate philanthropy has provided funding for both research and programme implementation. Drug donation programmes have provided funding for mapping, morbidity management, MDA implementation, monitoring, and operational research. And NGDOs that previously were dedicated only to eye care have joined the fight against LF (and other NTDs).

Dr Hopkins highlighted several examples of partnership and collaboration between Merck & Co., Inc. and GSK. They have worked to develop a joint drug application form, coordinate drug approval and delivery, and support a joint technical advisory committee. Partnership among NGDOs has been extraordinarily important to the GPELF. In 2004, the NGDO LF network was formed and in 2009 it joined with other groups to create the NGDO NTD network http://www.ntd-ngdonetwork.org.

Dr Hopkins pointed out that the challenges ahead will require new partnerships. It will be important to maintain the interest and commitment of existing partners; put greater priority on morbidity management; and implement effective post-MDA surveillance, which may not be as interesting to donors as MDA, but which is needed if we are to reach our goal.

#### Lessons Learned

Mr Andy Wright, Director of the LF Elimination Programme at GSK, began his talk by recalling that, when the GAELF was established, it resolved to "learn by doing." He then highlighted some of the lessons that have been learned during the last 10 years.

##### MDA

The current two-drug treatment strategy has reduced the prevalence of microfilaremia to < 1% in many settings. Community mobilization has been crucial to achieving the coverage necessary for success. It is also clear that five years of MDA will not stop transmission in all settings, and that additional or enhanced interventions are needed in challenging areas (e.g., *Loa loa*-endemic countries and urban settings).

##### Science

Mr Wright emphasized the important role played by science in guiding strategy, assessing drug efficacy, monitoring and evaluation, and determining the number of annual rounds of MDA required to stop LF transmission.

##### Partnership

The open and inclusive nature of the GAELF, with its light, regional, and representative governance structure, has worked well. The GAELF has matured as a partnership, and this has been facilitated by transparency and communication among partners.

##### Fund-raising

Several lessons related to fund-raising have been learned, including the importance of engagement by Ministries of Health; the role of international advocacy; and the attractiveness to donors of an integrated programme of NTD control.

##### Morbidity management

Although morbidity management has scaled up more slowly than MDA, it is a vital component of the GPELF that can enhance MDA uptake [11]. The effectiveness of basic lymphoedema management has been demonstrated in several settings; it can decrease ADL incidence, reduce lymphoedema progression, improve quality of life, and enhance economic productivity [7].

## Wednesday, 2 June, 2010

### Half-time Scores in Morbidity Control

**Chair**: Professor Dato C.P. Ramachandran

#### Impact of MDA on Clinical Disease

Charles Mackenzie, Professor of Parasitology and Diagnostic Investigation at Michigan State University, observed that accurate data are not available on the number of people affected with various forms of LF-related disease, and this limits our capacity to plan for and provide adequate care.

Since it began in 2000, the GAELF has continued to learn about the impact of MDA on clinical disease. Data from Tanzania indicate that MDA dramatically reduces the incidence of new cases of clinical disease. Dr Mackenzie argued that the incidence of clinical disease may be a critically important indicator of programme success. In Tanzania, MDA has been associated with reduced incidence, duration, and severity of ADL in persons with chronic morbidity [12]. Studies by Professor RK Shenoy, in India, also indicate that antifilarial drug treatment has an effect on sub-clinical disease in children (see below).

As the GAELF enters the next decade, Dr Mackenzie called for a clearer understanding of the extent and magnitude of clinical LF; more effective treatment, including improved wound and surgical care; and more widespread patient support systems, which have been piloted in Brazil, India, Tanzania, and elsewhere. He argued for improved funding and technical support for morbidity management, especially for specific patient needs, such as specialized shoes; assistance in job development and re-entry into the commercial community; psychological support; and comprehensive care packages. He stressed the need for more widespread innovation, such as the President Kikwete Fund, which supports hydrocele surgery in Tanzania.

#### Prevention of Lymphoedema in Children

Professor RK Shenoy, Chief of the Filariasis Research Unit at TD Medical College in Kerala, India described a study that he and his colleagues recently completed [13]. The study addressed the degree to which antifilarial drug treatment reverses clinical and sub-clinical disease associated with *Brugia malayi *infection in children 3-15 years old [14,15].

On enrolment, children were screened for microfilaremia, anti-*Bm *IgG4 antibodies, and clinical disease. Doppler ultrasonography and lymphoscintigrapy were done on all four limbs. Of 100 children enrolled, 32 were microfilaremic; 29 had clinical LF disease; and 39 had IgG4 antibodies indicative of *B. malayi *infection. All children were treated with a single-dose combination of DEC and albendazole and examined and re-treated every 6 months for 3 years.

A total of 18 adult worm "nests" were seen on ultrasound in 14 children, the youngest being 3 years old. Lymphatic vessel dilatation was detected by lymphoscintigraphy in 80% of enrolled subjects. At 36 months, lymphatic dilatation had reversed in 90% of affected children, and three of the four cases of clinical lymphoedema had resolved.

#### Integrated Morbidity Control: LF, Leprosy, and Diabetes Footcare

Dr Pierre Brantus, Consultant Physician to Handicap International, emphasized that strategies for morbidity management and disability prevention have to be based on scientific knowledge and yet implemented at the community level. The current WHO strategy for integrated morbidity management developed out of scientific research. It also is rooted in observations that the most consistent and lowest-cost care can be provided in the home, rather than an institution; that community-level programmes are most effective; and that psychological support is necessary for many patients. It is a comprehensive approach.

Dr Brantus noted several arguments for integrating LF morbidity management and disability prevention with care for other diseases. First, at the community level, the same health workers and general approaches are often shared, so integration can reduce costs. Second, fund-raising is often more successful for integrated programmes. Third, integrating LF elimination into NTD control programmes requires integration of morbidity management as well as MDA. Several disabling diseases could be integrated with LF disability prevention, including leprosy, diabetes, and Buruli ulcer, among others. Treatment for all these diseases involves hygiene, skin care, wound care, appropriate footwear, and movement. All could be addressed with similar home- and community-based approaches.

Mary-Jo Geyer, Professor of Health and Rehabilitation Sciences at the University of Pittsburgh, reported on the "Legs to Stand On" project. The first International Cross-Diseases Conference on Lower Limb Care in Developing Countries had been held recently in Accra, Ghana. Attendees included officials from international and national-level NGDOs, health professionals, patients, patient advocates, policy makers and programme managers, all with knowledge, responsibilities or expertise in LF, diabetes, leprosy, Buruli ulcer, and other lower limb conditions.

The goal of "Legs to Stand On" is to translate state-of-the-science evidence into cross-diseases curricula, educational materials, and programme guides for the implementation of lower limb care programmes to prevent disability in low-resource countries. The conference participants reviewed the current state of lower limb care programmes internationally and they used a flexible and comprehensive group decision-making method that was representative of all stakeholders.

The participants drafted detailed "problem statements" for several key issues (e.g., footwear), which addressed current needs, requirements, and specifications. The conference organizers are planning to hold a consensus conference in the fall of 2010 and to begin producing and using standardized technical tools in 2011.

#### Hydrocele Surgery

Dr Serigne Magueye Gueye, Professor and Chair of Urology, University of Cheikh Anta in Dakar, Senegal, updated the GAELF on the West African LF Morbidity Management Programme, which helps to train and equip surgeons to repair hydrocele, the most common chronic manifestation of bancroftian filariasis. He explained why surgery that spares the hydrocele sac may result in suboptimal outcomes in LF-endemic areas, and summarized key points for hydrocele surgery recommended by the programme. These include proper pre-operative evaluation to exclude scrotal lymphoedema; the use of local anaesthesia; an approach that uses a midline incision; meticulous haemostasis; proper post-operative dressing and bandaging; and complete resection of the hydrocele sac [16]. The West Africa LF Morbidity Management Programme has had considerable success. Some 3874 surgeries were performed during training courses, which have taken place in 11 countries. 415 health workers have been trained, and the work has been highlighted at major international urology meetings. The programme also provided training in connection with the President Kikwete Fund for hydrocele surgery in Tanzania.

To expand access to surgery for men with hydrocele in LF-endemic areas, it will be necessary to reposition LF within national health plans and to increase training and research through a network of public and private partners, including universities, United Nations (UN) agencies, and NGDOs. In conclusion, Professor Gueye stressed that hydrocele surgery can be done even in remote areas, as long as training is adequate. He called for the establishment of a broader network for morbidity management and training, as well as a GAELF Morbidity Management Expert Group.

#### Economic and Psychosocial Impact of Hydrocele and the Benefits of Hydrocelectomy

Professor John Gyapong, Director, Research Development Division, Ghana Health Service, presented preliminary results of a study now underway in Ghana, where hydrocele is a public health problem. In 2006, 9,931 cases of hydrocele were registered in the country. Several studies have shown the negative effect of hydrocele on productivity and quality of life, but little attention has been given to how these factors change following surgical repair of hydrocele (hydrocelectomy).

In the study, 215 men were interviewed before undergoing hydrocelectomy and 3, 6, 12, and 18 months after the operation to assess productivity, quality of life, cost of treatment, and clinical status. Beliefs about the cause of hydrocele varied, but few men considered it to be associated with a mosquito-borne parasite. Only 15% of men had ever sought care for hydrocele; for those who did, treatment consisted primarily of herbal preparations or puncturing the scrotal skin with a hollow reed to drain the fluid. Twelve months after hydrocelectomy, 85% of men were considered to be in "perfect health" based on several indicators including mobility, self-care, performance of usual activities, pain or discomfort, and anxiety/depression. The men also reported improved economic status and family life. Analysis of the data is ongoing.

#### Integrative Self-care through Community Participation for Morbidity Management

Mr Naveen Krishna Tarur, of the Institute of Applied Dermatology (IAD) and Infosys Tech Ltd., described an integrative self-care programme for lymphoedema management in Kerala, India [17,18]. He argued that lymphoedema treatment should incorporate self-care and be low-cost, locally available, and administered at the community level. Naveen acknowledged, with thanks, that the Government of India's Department of AYUSH (Ayurveda Yoga, Unani, Siddha, and Homoeopathy) has sponsored community-level morbidity management for 1,000 poor patients in two filariasis-endemic districts of India, based on IAD's integrated treatment model. This project is ongoing. He also called for a country-wide programme for India, which could be rolled out at the community level. The programme advocated by the IAD integrates the principles of Ayurvedic medicine; western biomedicine; Yoga (Pranayama); traditional skin care; and patient counselling and education.

##### Discussion

An animated discussion followed these presentations, which touched on the evidence (and lack thereof) for the effectiveness of various treatments for lymphoedema; the most effective components of existing treatment packages; the role of surgery; the best indicators of clinical and sub-clinical improvement; the lessons that have been learned from programmes for managing other chronic diseases, such as leprosy; and the importance of patient education. The varying degree to which these diseases are stigmatized in different settings may pose certain challenges for integration, especially for support groups. However, recent experience in Indonesia suggests that leprosy patients in mixed support groups progressed better than those participating in leprosy-only support groups (Professor Geyer, personal communication).

### Half-time Strategy: Future Research and Application

**Chair**: Dr Eric Ottesen

#### Strategies for the End Game: Operational Research Update

Dr Dominique Kyelem, Program Director at the LF Support Center, Task Force for Global Health, provided an update on operational research that will inform strategies for the "end game" - especially for post-MDA surveillance to detect possible resurgence in LF transmission. It is accepted as a "working hypothesis" that transmission will be interrupted when microfilaremia prevalence declines to < 1%, based on the experience of China.

The current Bill & Melinda Gates Foundation grant to the GAELF for operational research is focused on developing improved tools and sampling strategies for post-MDA surveillance. A guide for programme managers is being developed to assist them with survey methods and sample size calculations for post-MDA surveillance. The guide will help programme managers determine the "critical value" of positive tests among children 6-7 years old, above which MDA should be continued. The role of xenomonitoring in areas with *Culex*-transmitted filariasis is also being explored with support from this grant.

Operational research and programme experience indicate that, when five years of MDA are insufficient to reduce microfilaremia to levels that will not sustain further transmission, several factors may be in play. These include high prevalence and density of pre-MDA microfilaremia, low drug coverage or compliance, characteristics of the local vector, the drug regimen used, and other factors, such as the lack of social cohesion, especially in urban environments. In such situations, alternative strategies may be needed, including modified drug regimens (e.g., biannual MDA), vector control measures, or perhaps antibiotic treatment. Dr Kyelem emphasized that LF elimination is feasible, even in areas with the greatest challenges, but that continued operational research will be necessary for providing guidance to the GPELF [19].

#### Diagnostics: Development of Applicable Tools

Professor Gary Weil, Professor of Medicine at Washington University School of Medicine, emphasized the need for practical diagnostic tests, and noted the important role played by the antigen-detection based ICT card test for mapping LF-endemic areas in many parts of the world. He reported findings from a study in Egypt, where, with 5 rounds of MDA, the prevalence of antifilarial antibody (*Bm*14 IgG4) decreased more dramatically in children 6-10 years old than did antigen or microfilaria levels [20]. The prevalence and incidence of these markers decreased with each consecutive MDA. Professor Weil proposed target levels of microfilaremia, antigenemia, and antibody levels for programme decision-making. He noted the need for better and less costly tests; the lack of firm consensus on programmatic endpoints; the paucity of resources for monitoring and evaluation; and the need for applied field research to optimize the utility of existing and newly developed tests.

#### Role of Vector Sampling - Xenodiagnosis in Post-MDA Surveillance

Dr Sandra Laney, Research Scientist at Smith College, reviewed the advantages of xenodiagnosis, i.e., detection of infection in the mosquito vector, to monitor levels of LF infection in the community. There are two major approaches to xenodiagnosis: dissection of the vector to detect the parasite; and molecular xenodiagnosis (MX), using polymerase chain reaction (PCR) techniques. Current MX detects parasite DNA or RNA in the mosquito and can distinguish among filarial species. Dissection is less sensitive than MX.

Xenodiagnosis can help determine whether infection is present; it can serve as an indicator of transmission potential; and it can detect changes in transmission. It is less intrusive and more acceptable to the community than blood collection. Field research in the context of LF elimination programmes in Papua New Guinea, Egypt, and Tanzania has documented declines in mosquito infection rates with consecutive rounds of MDA [20].

More research is needed to make MX fully programmatically applicable. MX data may be hard to interpret programmatically in settings where vector control has been implemented, e.g., where bednet coverage is high or indoor residual spraying of insecticides is used. Still unknown is the effectiveness of MX over wide geographic areas; sampling strategies and sample sizes across regions; and how data on human infection correlates with MX. Studies are underway to assess the potential of xenomonitoring to detect resurgence of transmission following cessation of MDA, as has been used in onchocerciasis programmes in Africa. Additional work is needed to simplify sampling strategies, reduce cost, and develop better methods for mosquito collection and for use of PCR in the field.

#### Post-treatment Surveillance for LF: Togo Case Description

Dr Yao Sodahlon, Associate Director, Mectizan Donation Program, described a comprehensive system for post-MDA surveillance that has been established in Togo, where mapping with ICT in 2000 had indicated that LF was endemic in 7 districts. After 10 years of MDA (7 years with full geographic coverage), monitoring data suggested that transmission had been interrupted, so MDA was halted and post-MDA surveillance was initiated. The goal of this surveillance is to ensure that there is no ongoing LF transmission and to prevent re-introduction through prompt case detection and response. The surveillance system is low-cost and has been integrated as much as possible with other health activities. It has several components.

First, areas that were excluded from MDA on the basis of initial mapping are being re-mapped in 2010, using the RAGFIL method with a finer grid (e.g., 10 or 35 km) than was used initially (50 km). Second, surveys were done in two districts in 2009 to determine whether to stop MDA; these provided a baseline for post-MDA surveillance. In these surveys, 1548 school children 6-7 years old were tested by ICT; 2 (0.1%) ICT-positive cases were detected, one who was microfilaremic. Screening for microfilaremia around the index case revealed no infection. Screening will be repeated in 2011.

Third, laboratory-based surveillance was started in 2006, involving 40 laboratories that examine thick smears for malaria. All malaria slides that are collected at night also are read for microfilaria. In 2010, this system was evaluated. Some 4000 slides are read each year. Two microfilaria-positive slides have been detected, one from a nomad who was lost to follow-up and the other from a person living in a district considered non-endemic for LF. Finally, plans are being developed to test donated blood at blood banks using the ICT or the Og4C3 ELISA. However, the geographic distribution of blood donors is not geographically representative, so this approach may require further evaluation.

The second objective of post-MDA surveillance is to prevent recurrence or reintroduction of infection. This has been achieved by intensified mapping and laboratory-based surveillance in areas that border other areas of high-risk (e.g., other LF-endemic countries). The response to any ICT-positive result is to re-test that person for microfilaremia and, if positive, to treat. An epidemiologic assessment is made to determine if the case is local or imported. Community surveys will be done in areas suspected of being the source of infection, and MDA will be re-started if necessary.

#### LF and NTDs - The Chicken or the Egg: How do we Stop MDA?

Dr John Gyapong invited participants to recall the WHA resolution 50.29, which urged member states to undertake four key actions: 1) Take advantage of recent advances in understanding LF and its control; 2) strengthen local LF programmes and their integration with the control of other diseases, particularly at the community level; 3) strengthen training and capacity for research, management, and laboratory diagnosis; and 4) mobilize support from all relevant sectors.

In an integrated programme, how can we stop MDA for LF when there is "unfinished business" for other NTDs? Dr Gyapong pointed to several factors that contribute to a gap between knowledge and action, the "know-do" gap. These include the complexity of integrated programmes; inadequate evidence and data for decision-making; challenges with existing guidelines; and inadequate funding for monitoring and evaluation activities. Dr Gyapong suggested several practical issues to consider, including the feasibility of joint monitoring and evaluation for preventive chemotherapy; equipping country programme managers to adequately monitor and evaluate their interventions; and improving the capacity for preventive chemotherapy programmes to collect the minimal data needed for the global programme. He noted that the needs for epidemiological assessment may differ among NTDs but that it's possible to plan so that data can be pulled together to make informed and integrated decisions [21,22].

Dr Gyapong argued that an integrated NTD control programme should not be integrated only at the level of programme implementation and advocacy. Rather, decisions about stopping MDA also should be made in an integrated context, and this should be based on integrated monitoring and evaluation data. He challenged the GAELF to move from monitoring processes to assessing impact, and urged that the GAELF come to consensus on what will be needed to achieve this.

##### Discussion

In the discussion that followed, Dr Ottesen highlighted the important role of research in the GPELF, and pointed to the research now underway to refine guidelines for stopping MDA and to fit these guidelines into the "new world of NTDs." Comments were made by several participants on the recently-developed rapid test for *Brugia *infection and its performance in the field. Discussion also addressed xenomonitoring and different methods of mosquito collection.

### Half-time Strategy: Major Technical Challenges

**Chair**: Dr Frank Richards

#### LF in the City - The Urban Problem

Dr Margaret Gyapong, Director of the Dodowa Health Research Centre, reviewed the challenges presented by rapid urbanization. Some 38% of Africans live in urban areas, and MDA coverage in urban areas in Africa has been sub-optimal (generally 40-50%). Contributing to this is the fact that people who live in cities tend to be busier, making social mobilization more difficult; populations are heterogeneous, with complex social, economic, and religious structures; and urban dwellers place a higher priority on privacy. In urban areas, communities tend to be defined by affiliation or identity, rather than by geographical proximity. Because of these differences, simply importing MDA strategies from rural to urban areas is not likely to be successful.

Specific challenges to MDA in urban areas begin with defining and demarcating the community; slums are often immediately adjacent to the high-rise apartments of some of the richest - and certainly the "non-poor". Community-directed treatment (ComDT) and use of volunteer distributors does not work as well in urban areas. Elites, who may be at risk of LF in urban areas, perceive their risk as being low, consider LF a "disease of the poor," and limit access through security guards and dogs. In such a setting, what is the appropriate denominator for calculating drug coverage?

However, with appropriate preparatory work these problems can be addressed. Populations can be characterized not only by location but also by socioeconomic, religious, and demographic status. Existing informal networks can contribute to MDA implementation. Knowledge of existing health and related interventions can be helpful. Regardless of how the community is defined, it needs to be engaged and consulted to determine the best approaches. It is important to involve community members, to seek their input and suggestions as collaborators, and to empower them to make decisions and implement and manage change.

Urban MDA will require more involvement of the private sector than is typically the case in rural areas. This includes private medical practitioners; hospitals; private clinics; other non-health sectors; politicians; and others. A team approach should be used, with as many stakeholders as possible, for advocacy planning.

##### Discussion

In the discussion that followed, Dr Frank Richards suggested the label "white coat environments" for urban areas, because urban dwellers prefer to take medication only from "professionals" wearing white coats. Professor Molyneux raised the question as to whether *Culex *in West Africa may be genetically insusceptible to infection with *W. bancrofti *- which would have significant implications for urban transmission, and this was discussed further.

#### LF in the Forest

Dr Joseph Kamgno, Director of the Filariasis Research Centre in Cameroon, reviewed the challenges posed by co-endemic LF and *Loa loa *infection in Central Africa. In the forested areas of these countries, serious adverse events (SAEs) following treatment with ivermectin have been a major obstacle to expansion of both onchocerciasis control and LF elimination programmes. The main risk factor for SAEs is high density of *Loa loa *microfilaria in the blood (especially above 10,000 per mL) [23].

Operational measures, including surveillance, have been implemented at regional and community levels to reduce the frequency and improve outcomes of persons with SAEs. In LF-endemic areas of Central Africa where MDA with ivermectin for onchocerciasis has already been implemented, the risk of SAEs is low; MDA can be continued and albendazole co-administered for LF elimination. In areas where no MDA has yet taken place, research is underway to determine if twice-yearly treatment with albendazole alone can sufficiently reduce *Loa loa *microfilarial densities to safe levels. Insecticide-treated bednets also may be useful. Research is ongoing to assess cofactors associated with SAEs, including *Loa loa *strain differences and human genetic factors. Programmatic approaches also are being evaluated, e.g., the feasibility of test-and-treat approaches, and the impact of limiting MDA to younger people at lower risk of SAEs. Detailed mapping of *Loa*-endemic areas in Central Africa continues.

##### Discussion

The ensuing discussion focused on the pathophysiology and treatment of SAEs, on the need for accelerated research, and on various strategies to overcome the challenges posed by *Loa loa *for LF elimination in Central Africa.

#### LF in Conflict Zones

Dr Adrian Hopkins reflected on the specific challenges to LF elimination in zones of conflict. He noted that, in most conflict zones, only a small percentage of the population is actively engaged in fighting. Once fighting has erupted, the short-term needs of the population are for food, water, and shelter. Health concerns are a longer-term priority, although health quickly becomes a priority in refugee camps, which can be quite organized. The special challenges of working in conflict situations include the risk of violence; destruction of infrastructure, medical records, and research data; reluctance to make any further investment in infrastructure, offices, schools, or hospitals; and shortages of human resources, such as well-trained staff.

Even with these challenges, however, MDA can be successful in such settings. For example, onchocerciasis programmes persisted and even expanded during periods of conflict in the Central African Republic, Sudan, and the Democratic Republic of the Congo.

Lessons from these experiences include the importance of investing in communities, which can be quite resilient; the need for flexibility and mobility; the increased cost of doing business in zones of conflict; and the need for appropriate infrastructure (e.g., laptop computers rather than desktops). With adherence to these principles, MDA can be realistic for many areas in conflict [24,25].

#### LF after MDA

Dr Mwele Malecela noted that strategies for stopping MDA and initiating post-MDA surveillance still need to be fine-tuned. However, the programmatic benefits of LF elimination will persist even after LF has been eliminated. Other NTDs will likely remain, and the infrastructure that was developed to eliminate LF can be transformed for use with other NTDs. Similarly, the benefits of strengthening the health system for LF elimination will persist. These benefits include human resources; controls and procedures for managing the drug inventory; recording and reporting systems; and cascade training programmes. Community-based distributors trained for LF might well play a role as *bona-fide *health workers in an expanded health system.

Morbidity management activities will continue, including patient support and advocacy groups and home-based health care for lymphoedema, preferably integrated with care for other non-communicable diseases. The need for hydrocelectomy will continue, as will the need for psychological counselling (many men with hydrocele in the recent "hydrocelectomy camps" in Tanzania reported being suicidal). The President Kikwete Fund for hydrocele surgery was begun in response to awareness of the magnitude of the problem - more than 15,000 affected men - which called for action.

In conclusion, Dr Malecela reiterated that the patient remains central to the LF programme, and urged programme managers in the next decade to focus on surveillance.

##### Discussion

Several topics were addressed in the discussion, including the advantages and disadvantages of using cellphones for surveillance, patient support groups, provision of mental health services, and timely notification of health workers regarding ADL episodes in patients with lymphoedema.

### Half-time Strategy: Alternative Strategies for the Second Half

**Chair**: Professor Moses Bockarie

#### Filariasis Chemotherapy for the Next Decade

Professor Gary Weil reviewed the history of drug therapy for LF, beginning in 1947 with DEC. While two-drug combinations given in a single annual dose are the mainstay of LF elimination, they have some limitations. They are not completely effective against the adult worm; they are associated with adverse events; and they cannot safely be used in areas with intense *Loa loa *transmission. Lack of compliance is an issue with current approaches to MDA; a recent study in Egypt by El-Setouhy and colleagues showed that 7% of people had never taken the drug after 5 rounds of MDA [26]. The potential for drug resistance is also a concern. Although resistance to ivermectin and albendazole has not yet been observed in LF parasites, few data exist or are being collected.

Professor Weil summarized some of the current and planned research on filariasis chemotherapy. Recent data suggest that repeated doses of albendazole alone are safe in *Loa*-endemic areas, and may be fairly effective in reducing *W. bancrofti *microfilaremia. Phase 3 clinical trials are currently testing moxidectin, a drug similar in structure to ivermectin. A multi-faceted group of studies, known collectively as Death to Onchocerciasis and Lymphatic Filariasis (DOLF), is now underway, funded by the Bill & Melinda Gates Foundation. Large-scale community trials of biannual "enhanced" MDA will include epidemiologic modelling and cost analyses (Peter Fischer, Principal Investigator [PI]). Randomized clinical trials will assess the efficacy of new drug combinations and treatment schedules (James Kazura, PI), and flubendazole is being tested in pre-clinical studies for its potential as a macrofilaricidal drug (Charles Mackenzie, PI).

Professor Weil suggested that more frequent MDAs over a shorter period could be more effective, reduce overall costs and programme duration, and decrease the likelihood of drug resistance.

#### Role of Antibiotics Against *Wolbachia*: Can it Play a Role in the Endgame?

Mark Taylor, Professor of Parasitology at the Liverpool School of Tropical Medicine, spoke about the potential role of antibiotics against *Wolbachia*, symbiotic bacteria that are found in *W. bancrofti, B. malayi *and *Onchocerca volvulus*. Treatment with doxycycline eliminates *Wolbachia *from these parasites, leading to permanent sterilization, sustained loss of microfilaraemia and potent macrofilaricidal activity. Studies in patients with lymphoedema who were treated with doxycycline have shown reductions in lymphatic vessel diameter and lymphoedema severity, as well as improvement in skin condition [27]. Treatment with doxycycline also reduces severity of hydrocele in men who are actively infected with *W. bancrofti *[28].

A 4-8 week course of doxycycline is highly effective and well-tolerated, but the logistics of delivering long courses of treatment and contraindications in children and during pregnancy are barriers to the widespread introduction of doxycycline for MDA. However, Wanji and colleagues recently completed a study of community-directed delivery of a 6-week course of doxycycline in an area co-endemic for onchocerciasis and loiasis, in which compliance was 98% and therapeutic coverage was 74%, demonstrating that in more restricted areas this option is both feasible and achievable [29].

Professor Taylor described the Anti-Wolbachia Consortium (AWOL), a five-year, $23 million research programme funded by the Bill & Melinda Gates Foundation to find new anti-*Wolbachia *treatments compatible with community-treatment programmes for human filariasis. Activities include refining current regimens that use doxycycline; developing assays to rapidly screen and test new drugs that may be even more effective than doxycycline; studies to better understand the role of *Wolbachia *in filarial worms; and identifying the genes that are essential for the organism's survival.

#### Role of Vector Control

Professor Moses Bockarie noted that LF is the only vector-borne disease that is transmitted by more than 4 genera of mosquitoes, each with different features and capacities for transmission. The good news for LF elimination is that, even in areas with the most efficient vectors, MDA alone can interrupt LF transmission using two-drug combinations. However, 19 countries with active LF transmission have not yet begun MDA. Of these, 13 have fragile infrastructures or are in post-conflict situations; six others are stable, but with low LF endemicity. In 17 of these countries, *Anopheles *is the principle vector for LF [30].

This is good news for LF elimination. The efficiency with which *Anopheles *transmits LF is low compared to other vector species, and *Anopheles *also is the principle vector for malaria. Early experience with DDT house-spraying in the Solomon Islands, Papua New Guinea, Togo, and Indonesia showed dramatic effects on LF transmission [30], and insecticide-treated bednets (ITNs) are even more effective than DDT as a malaria control strategy. A study in Liberia and one currently underway in Nigeria have documented the effectiveness of ITNs in reducing the density of LF infection in mosquitoes under conditions of universal bednet coverage [31]. Together, these data suggest that ITNs can have significant impact on LF transmission.

Thus, Professor Bockarie suggested that in these 17 countries the LF elimination strategy should not be "MDA and vector control if possible" but, rather, "MDA and vector control." ITNs are being widely distributed for malaria control, so there is reason for hope.

##### Discussion

An animated discussion followed that touched on the role of vector control and the strength of scientific evidence that ITNs reduce LF transmission, especially within national programmes. Other comments focused on the feasibility and effectiveness of twice-yearly albendazole in *Loa-loa *co-endemic areas and the advantages and disadvantages of various recommendations put forward by the presenters, particularly the feasibility of incorporating antibiotic treatment into operational programmes.

## Thursday 3 June, 2010

### Second Half 2010-2020: Strategy for the Next Decade

**Chair**: Dr Dirk Engels

#### The Vision Moving Forward

Dr Dirk Engels presented WHO's vision for LF elimination in the context of an integrated, multi-disease approach. He reviewed the major challenges ahead and suggested action points for each one.

##### Getting started

MDA should be initiated in the 19 countries that require it but have not yet started. Of these, 16 are located in Africa [1].

##### Loa loa

In Central Africa, *Loa loa *co-endemicity presents a major barrier to initiating LF elimination programmes. Research is urgently needed to find alternative or provisional strategies.

##### Upscaling

In the countries where MDA has already begun, it is critical to upscale MDA to full geographic coverage. 70% of the total at-risk target population, 919.5 million people, live in the countries of India, Indonesia, Bangladesh, Nigeria, and the Democratic Republic of Congo. Achieving and maintaining full geographic coverage is particularly important in these countries.

##### Urban populations

Strategies must be developed to effectively treat urban populations where this is needed, particularly in Africa and Asia.

##### Disability management

Only 27 countries have active disability management programmes; this needs to be expanded and linked to other disability management programmes (e.g., for trachoma or leprosy). It should include home-based counselling and strengthening of the health system through improved NTD case management.

##### Guidelines

An urgent need exists for refined guidelines for stopping MDA and for post-MDA surveillance. Such LF-specific procedures and guidelines are a priority for WHO. However, these guidelines should be part of an integrated monitoring and evaluation package; MDA coverage will be the basic indicator, but the guidelines will also include other disease-specific indicators.

##### Integration

In addition to integrated monitoring and evaluation, WHO envisions an integrated approach to three key areas: preventive chemotherapy, disability management, and vector control.

**Figure 5:**
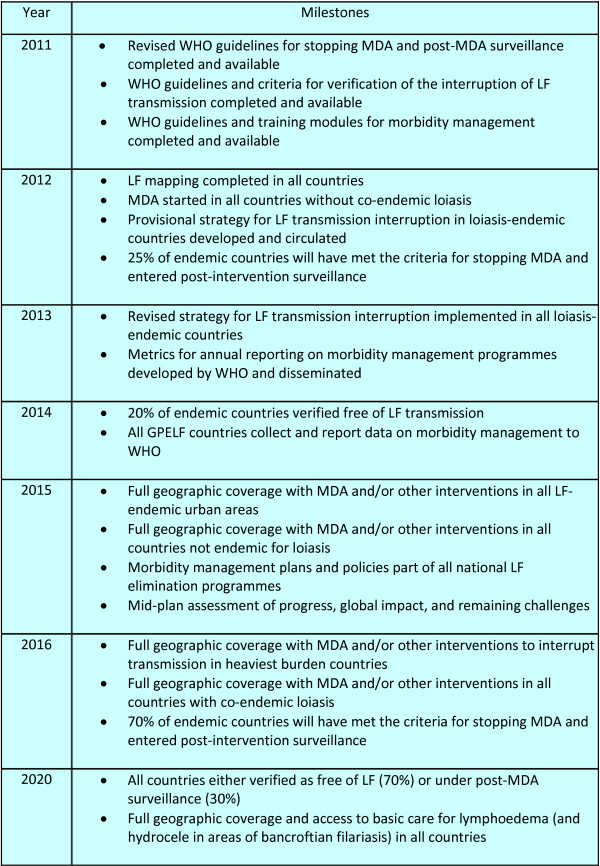
**Milestones in a draft strategic plan for LF elimination proposed by the World Health Organization (WHO)**.

Dr Engels presented milestones proposed by WHO in a draft strategic plan for the GPELF (Figure 5), all of which lead to the goal that all LF-endemic countries will either be verified free of LF or under post-MDA surveillance by 2020. He stressed the need for harmonizing the language used in the GPELF with that of other NTDs, and suggested that the phrase "elimination as a public health problem" be interpreted as meaning prevention of morbidity.

The public health problem to which WHA resolution 50.29 refers has to do with morbidity, not just transmission of the parasite. One articulation of a morbidity-related goal for 2020 might be, "To provide access to preventive treatment and care for every individual living in LF-endemic areas, through an integrated approach."

#### Building Partnerships for Morbidity Control

Dr Pierre Brantus cited the need to develop partnerships to improve implementation of morbidity management and disability prevention within the GPELF. He defined partnership as a relationship between individuals, groups, diseases or health problems that is characterized by mutual cooperation, responsibility, and interaction for the achievement of a specified goal.

Dr Brantus outlined the nature of various partnerships. Some partnerships develop out of a desire to combine or integrate *activities*. Within the GPELF, examples of these include partnerships between MDA and disability prevention activities, between wound care and lymphoedema management, and among the various programmes that focus on LF, leprosy, diabetes, or Buruli ulcer. Other partnerships are established primarily to combine and share *resources*. Examples include partnerships among NGDOs; between scientific institutions and organizations focused on operations or delivery of services; and between Ministries of Health and NGDOs.

Major challenges for partnerships in morbidity management and disability prevention include: improving involvement and collaboration at the country level; modifying the way donors fund projects and the rules for managing partnerships; and developing new partnerships to address the problem of hydrocele, which will require recruitment of new donors and organizations involved in promoting reproductive health [8].

#### Building Partnerships for Implementation

Professor Bernhard Liese, Chair, International Health Department, Georgetown University, commented on the diverse nature of the GAELF partnership, which involves LF-endemic country governments; drug donation programmes; multilateral and bilateral donors; private foundations; and NGDOs.

The central issue for the next decade of the GAELF is access to funding that will allow programmes to go to scale. The key constraint to scaling up has been lack of funding rather than technical issues, governance, or inadequate operational research. Both the availability and the stability of funds are needed.

From the perspective of funding, the contribution of various partners within GAELF varies considerably. Official development assistance from donor governments for health contributes only 0.6% of all funding for NTDs [32]. In contrast, the drug donation programmes have been major and reliable partners, in part because there is no *replacement effect *related to their donation. In other words, Ministries of Finance of LF-endemic countries do not reallocate their health budgets to other sectors in response to drugs donated to the health sector, whereas they often do so if direct funding for other health activities is received from donor governments.

To increase the level of funding, donors should consider pooled-financing mechanisms, such as a Trust Fund at the World Bank. The Trust Fund of the African Programme for Onchocerciasis Control (APOC) is one such example. Advantages of such mechanisms include the fact that the funds are usually non-designated, and therefore flexible; they are generally stable; and it is easier for international development banks to provide funding. Challenges include the need to clarify governance issues and the role of the secretariat.

Within LF-endemic countries, both the Ministry of Health and the Ministry of Finance should treat LF as an "essential expenditure" line item in the national budget. Potential partners within the national government are often overlooked, such as the national health insurance system and hospital network. Morbidity management has been the most neglected part of LF elimination. To develop, it should be viewed within the context of health system strengthening. NGDO support for morbidity management will be critical for the next decade.

#### Developing the Operational Research Agenda

Dr Julie Jacobson, Senior Program Officer, Bill & Melinda Gates Foundation, reviewed some of the operational research projects supported by the Foundation. She argued that history is filled with failed attempts at disease elimination or eradication, and that these failures can provide important lessons. For example, eradication of yellow fever was attempted without the research necessary to recognize the importance of non-human reservoirs. She urged the GAELF6 participants to ask the "hard questions." Are we willing to fail? If not, what must we put in place to meet our targets? Why have some LF programmes not succeeded in interrupting transmission after 5-9 years of MDA? How will the problem of co-endemic loiasis be addressed in Central Africa? What are our strategies for urban MDA and for post-conflict and conflict settings? What will be the impact on LF of integration with other NTD programmes, and how do we ensure that integration helps the cause of LF elimination?

Several challenges present themselves for the next decade of the GPELF. Countries that remain highly endemic for LF are precisely those that have the least experience with LF. Modified strategies and thresholds must be developed as different approaches are put into play. Conflict and post-conflict situations, the perceived low priority of LF in the remaining countries, and the need for clear guidelines represent additional challenges.

##### Discussion

An enthusiastic discussion followed on the importance of operational research; the intent of the original WHA resolution; and the role of morbidity management, including the fact that it has not progressed as quickly as MDA. Several speakers and participants noted that the focus on integration provides a unique opportunity to learn from other health programmes such as those addressing leprosy, obstetric fistula, or malaria.

Dr Engels suggested that the next WHO 10-year strategic plan be considered in two 5-year periods. During the first period, the emphasis will be on implementing and upscaling MDA in all countries, and on stopping MDA and initiating surveillance in areas where that is possible.

#### African Development Bank

Dr Tshinko B. Ilunga, Manager of the Health Division, African Development Bank (ADB), described the Bank's health programmes, which assist regional member countries in addressing health problems, implementing health policies, and strengthening the health system. It also promotes investment in other sectors that have a direct bearing on health improvement (e.g., water and sanitation).

The focus of the ADB's on-going projects and programmes include public health promotion and health systems strengthening (through formulation of health policies and strategies; introducing reforms; and building capacity through training and infrastructure). Within the last five years, priority areas have included direct health investment through the public and private sectors and investing in environments that support health (e.g., food security, water and sanitation, communication infrastructure).

Currently the health portfolio of the ADB is estimated at $690 million, with 33 active investment projects in 30 regional member countries. The potential for greater impact exists, as committed resources are not fully utilized and countries tend not to request funding for health. Dr Ilunga emphasized that all available resources will be needed to ensure that LF elimination is funded at an adequate level.

#### USAID NTD Strategy 2010-2014

Ms Angela Weaver, NTD Advisor for USAID, described the agency's programme to address NTDs. This programme began in 2006, when the US Congress approved special "earmark" funding of $15 million per year. USAID awarded a competitive agreement to RTI to issue competitive grants to leading technical partners; support upscaling of MDA; help recipient countries gain access to donated NTD drugs; contribute to lessons learned and best practices; and develop state-of-the-art tools for monitoring. The project started in 5 countries (Burkina Faso, Ghana, Niger, Mali, Uganda - so-called "fast track" countries), and has expanded through a competitive grant process to include eight additional countries. The focus of USAID support has been to upscale preventive chemotherapy. USAID agrees with the programmatic advantages of an "integrated approach" to preventive chemotherapy. However, it is also important to maintain a disease-specific focus; indeed, integrated preventive chemotherapy may open opportunities to accelerate disease-specific targets.

USAID funding for NTDs was increased to $25 million in fiscal year (FY) 2009 and $65 million in FY 2010. The goal of this investment is to expand integrated control of targeted NTDs to 30 countries, reducing the prevalence of these diseases by at least 50% among 70% of affected populations. An estimated 1 billion treatments will be provided, and it is hoped that onchocerciasis will be eliminated in the Americas and LF will be globally eliminated. USAID will continue to support integrated NTD control in the 14 countries already being supported and to expand its support to an additional 16 countries by 2014.

#### World Bank

Professor David Molyneux read a speech prepared by Professor Donald Bundy, currently the APOC Coordinator at the World Bank. Unfortunately, Dr Bundy was unable to attend the GAELF6. The Joint Action Forum (JAF) of APOC recently endorsed the need to expand integrated NTD activities including LF, and some APOC countries are currently implementing integrated programmes. This welcome news highlights the importance of evolving partnerships and the desire on all sides to seek new ways of working together.

#### Alternative Resource Strategies for NTDs

Dr Patrick Lammie, Technical Director for the Global Network for Neglected Tropical Diseases, highlighted the progress of the GAELF since its inception. The "spectacular" drug donations by Merck & Co., Inc. and GSK, as well as early support to GAELF from the UK Department for International Development (DFID) and the Bill & Melinda Gates Foundation, provided the critical sparks that launched the programme.

Despite these initial major contributions, further advocacy for LF alone was not terribly successful. In contrast, advocacy for NTDs has been remarkably effective. The Bill & Melinda Gates Foundation provided funding support for operational research on integrated control of NTDs and USAID followed with an initial $100 million in 2006. This was followed by the Obama Global Health Initiative, which represents the culmination of several years of concerted and targeted advocacy by many in the NTD community. The recently-announced USAID request for applications (RFA) would provide an additional $450 million for control of NTDs over the next five years. This signals a tremendous commitment - but even this amount will not suffice. It will be critical to mobilize additional resources if we are to finish the job of LF elimination.

The Global Network for NTDs is an advocacy and resource mobilization initiative working with international organizations, governments, technical agencies and donors to enhance collaboration and coordination in support of NTD control and elimination goals. Most of its support comes from the Bill & Melinda Gates Foundation. The Network works with WHO and other partners to promote the development of regional NTD control and elimination strategies; facilitate the development of coordination mechanisms in support of these strategies; and mobilize new resources to support country programmes. Because programmes and challenges differ from region to region, regional coordination mechanisms can build demand, improve resource flow, and support control efforts tailored to regional needs.

Dr Lammie presented details on the Latin America and Caribbean (LAC) Trust Fund Partnership, an example of a successful regional effort to address NTDs. The partnership includes the Global Network, the Inter-American Development Bank (IDB) and the Pan American Health Organization (PAHO), as well as private foundations and governments in affected countries. Early lessons learned from LAC include the need for strong partnerships and regional stakeholders; the essential role of governments; the advantages of advocating for integration of NTD control and elimination efforts with other initiatives, such as sanitation and housing; and the necessity of having good data to successfully mobilize resources.

Looking forward to the next 10 years, Dr Lammie made several points. The NTD message has been very effective, and it has provided a low-cost, high-impact intervention for the "bottom billion." The current global health landscape is both complex and competitive; donors are looking for integration and harmonisation on a much broader platform than NTDs alone. Successful advocacy is issue-oriented, tailored to the interests of specific donors and partners. Nothing is more important than morbidity management - yet efforts to address morbidity have lagged behind. We need new partnerships with organizations focused on integrated morbidity management.

Dr Lammie highlighted the need to address several issues related to programme integration, including NTD surveillance and vector control, the role of water and sanitation, and completion of NTD mapping. He encouraged the audience to share their stories of success and their data, and he concluded with a positive message: Advocacy works.

### Future of the GAELF Partnership

**Chair**: Mr Andy Wright

#### NGDO Linkages Between LF and Other NTD Groups - Simon Bush

Mr Simon Bush, Director of African Alliances and Advocacy for Sightsavers International, reviewed the role of NGDOs in NTD control. NGDOs tend to have relatively flexible organizational structures that can react quickly and adapt to country needs. They can provide support to country-level or global programmes; catalyse movement towards national "ownership" of programmes; bridge between the formal health system and communities; help broker relationships across sectors; develop effective delivery models that can be taken to scale; and help mobilize resources and advocacy.

In 1992, the NGDO Coordination Group for Onchocerciasis Control was established, followed by similar groups in support of trachoma and LF elimination. These groups began to meet together in 2006 to develop the NTD NGDO Network. The primary mission of this network is to coordinate activities of members and attempt to bridge gaps in funding. The network is not principally a fund-raising organization, but individual members continue to raise funds to support specific activities.

Mr Bush encouraged all delegates at the GAELF6 to advocate for inclusion of NTDs in the review of the MDGs, which is currently being undertaken. A UN review summit is scheduled for September 2010 in New York City.

#### Opportunities: Advocacy and Broader Partnerships in the Evolving Global Health Environment

Professor Molyneux noted that as late as 2000, the largest NGDOs involved in promoting eyesight and combatting blindness were not yet involved in LF elimination. Since then, they have joined forces with the GAELF - and in some cases, this has resulted in changes to their mission statements and governance. In "teaming up" with NTDs the LF community should consider several issues and questions. Should the GAELF continue as an entity - should the GAELF "brand" be maintained? How should the GAELF engage with groups representing other NTDs, and what mechanisms exist for such engagement? Should the GAELF focus its advocacy primarily on the large and epidemiologically important countries?

Several lessons have been learned in the short 10-year life of the GAELF. The capacity for programme implementation is a precious resource at all levels. Advocacy is essential. The new global health environment is complex and rapidly changing. It is impossible to predict exactly how global health will continue to develop, although the importance of non-communicable chronic diseases will continue to emerge. In 2015, we will enter a "post-MDG environment," with new challenges and opportunities. Professor Molyneux suggested that we keep the "NTD brand," with LF at its core, and highlight our successes for the purposes of advocacy.

Alternative strategies for LF elimination should be explored, while vigorously pursuing the current strategy. The application of alternative strategies raises many questions, including the possible role of antibiotics, and how they might be used in specific populations. Vector control should be enhanced, but where, how, and by whom?

##### Discussion

An animated discussion followed Professor Molyneux's presentation. Dr Richards agreed that the NTD "brand" is useful for advocacy, but pointed out that disease elimination also is a cause that has successfully attracted donors. He argued that we should not forget the goal of LF elimination, even within the context of NTDs. Dr Gyapong stated that use of antibiotics for LF elimination is a worthy topic for research, but premature as policy. Dr John Ehrenberg, from WPR, made the case for prioritizing a final push for LF elimination in the Pacific.

Dr Jacobson suggested three innovations for next 10 years. First, a "buddy" or partnership programme could be developed between countries just beginning LF elimination programmes and those with experience - especially south-to-south linkages. Second, more rapid streamlining of research results into the field could be facilitated by programme advisors, who could work with country programme managers to keep them abreast of latest research developments and provide consultation on implementation. Finally, she suggested an "LF elimination think-tank" to consider deeply and in detail what is needed to achieve the 2020 goal.

#### Conclusions and Reflections

Dr Mwele Malecela introduced the new Chair of the GAELF Representative Contact Group (RCG), Maged El-Setouhy, Professor of Public Health and Epidemiology at Ain Shams University, in Cairo. She also announced the results of the election for the Executive Group (see below).

Dr Malecela reflected on her 4-year term as Chair of the RCG. During this time, the RCG has worked to establish regional platforms that focus on regional issues and priorities in LF elimination. She emphasized that the regionalization process will continue with integrated NTD control programmes. She also noted that, with the growth of the GAELF, there has been some discussion about its future structure, and that this issue has been referred to the next GAELF meeting.

Dr Malecela reviewed the major developments in LF elimination over the past four years, concluding that "the strategy does work - the glass is half-full." Operational research has been critical in addressing challenges, and this will continue. She encouraged GAELF members to embrace integration with NTDs and to serve as leaders within the new initiative. Of the many challenges that were discussed during the GAELF6, upscaling MDA is "the big one." Concentrated efforts need to be made in morbidity management, with the help of new partnerships.

### Closing Ceremony

Dr Engels noted the progress made during the first 10 years of the GAELF, and agreed that the glass is half full. But, he noted, there is no room for complacency. Major challenges lie ahead, and strategies have been put in place to face the challenges that will arise. Dr Engels emphasized that teaming up with NTDs offers new opportunities for advancing LF elimination. He thanked the Korean hosts of the GAELF6, as well as Dr Malecela and Professor Molyneux, and pledged WHO support to Professor El-Setouhy and Dr Lammie in the months ahead. These thanks were echoed by Dr Malecela.

Professor Molyneux thanked Dr Lee, Professor Chai and Professor Rim for their enthusiastic support of this meeting. He praised the strong tradition of parasitology in Korea. He also acknowledged, with gratitude, the work of the GAELF secretariat in Liverpool.

Dr Jong-Koo Lee expressed his gratitude and thanks to several individuals and to all participants for a successful meeting. For a list of persons who attended, see additional file 1. Dr Lee thanked all those who attended for their commitment to eliminate LF and he expressed hope that the collaborative network represented in this GAELF meeting had been strengthened. He pledged Korea's continued participation in LF elimination through sharing its experience and providing technical support. Dr Lee thanked all the staff from KCDC for their work in preparing for GAELF6, and he declared the meeting closed.

### Business Session of the Representative Contact Group (RCG)

Dr Mwele Malecela called to order the business meeting of the RCG. Mrs Joan Fahy distributed ballots for new officers and elections were held. Ms Angela Weaver, of USAID filled the vacant position for donors. Georgetown University (Professor Bernhard Liese) and Michigan State University (Professor Charles Mackenzie) filled the two vacancies for academic and research institutions. Professor Maged El-Setouhy was elected Chair of the RCG. Executive Group members elected were: Patrick Lammie; Adrian Hopkins; Dominique Kyelem; Moses Bockarie; and Frank Richards. Two ex-officio country members, Doris Njomo (Kenya) and Rita Kusriastuti (Indonesia), were appointed. Dr Lammie was elected Chair of the Executive Group.

Professor David Molyneux thanked Dr Malecela for her leadership, time, energy, and thoroughness as Chair of the RCG. Dr Malecela expressed her pleasure in working with Professor Molyneux and with all the members of the RCG and the Executive Group, saying that it had been "a great honour and a great experience."

### Special Session: Enhancing Disability Prevention Implementation through Partnerships

***Chair***: Dr Pierre Brantus

A special session for NGDO representatives and other interested parties was held after the close of GAELF6. Focusing on how to develop partnerships to prevent LF disability, the session was co-chaired by Dr Pierre Brantus, from Handicap International, Professor Mary-Jo Geyer, from the University of Pittsburgh, and Mr Jose de la Cruz, from LEPRA.

**Figure 6:**
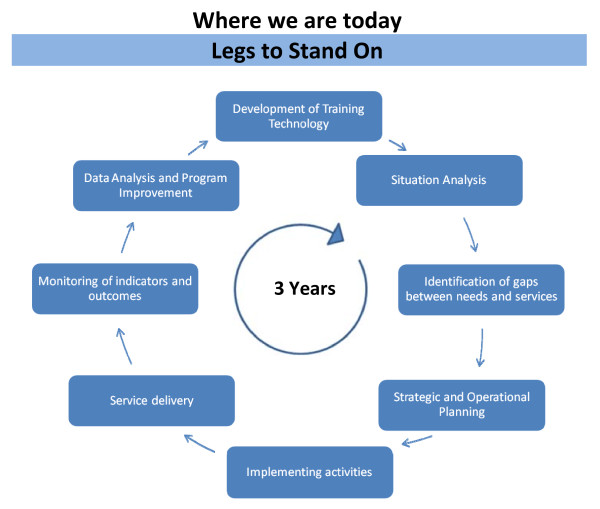
**Cycle of activities proposed in the "Legs to Stand On" project**.

Professor Geyer began by describing the "Legs to Stand On" project in greater detail. Priorities for the project include developing the training technology for cross-diseases morbidity management and conducting situation analyses in specific countries, followed by a series of planning, implementation, and evaluation steps (Figure 6).

Dr Brantus opened the discussion with the general question, "How are we going to work together?" Professor Ramachandran asked about specific guidelines for lymphoedema management. Dr Brantus indicated that WHO guidelines have been established for a "minimum package" that can be implemented in all LF-endemic areas, and that they soon will be published and distributed. In some areas, additional resources and treatment modalities, such as Ayurvedic and surgical approaches for specific cases, may be available. These are welcome, but they are not considered part of the minimum package for all LF-endemic areas.

Dr Manokaran Gurusamy suggested that several multi-disease treatment centres be established to support pilot research on the epidemiology, clinical spectrum, and treatment for the various conditions in which limb care is required. Professor Geyer noted that disability prevention centres for leprosy exist in many countries, and they already are treating people with diabetic feet. Additional diseases could readily be integrated. For any specific geographic area, it is important to do an initial stakeholder analysis to identify which organizations and NGDOs are available as potential partners. Dr Brantus agreed, emphasizing the need for careful identification and cultivation of partnerships before embarking on cross-diseases projects, especially if funding and other support is to be sustainable.

Dr Paul Maurice Dogbo Pepe, from Cote d'Ivoire, emphasized the need for better mapping of lymphoedema cases, as well as other lower limb conditions, as a necessary precursor to establishing treatment centres.

Mr Jose de la Cruz noted three types of unfamiliarity that create barriers to integrated management of lower limb conditions. First, there is lack of familiarity with the clinical and social tools to address these problems, for which guidelines are necessary. Second, there is a lack of knowledge about the epidemiology of various lower limb conditions in most areas. Third, there is a lack of familiarity with other organizations that are already addressing some of these conditions (e.g., NGDOs working in leprosy) that can be integrated with LF morbidity management. He suggested that NGDOs that are working in the field can provide WHO with a map that indicates they are active, what they are doing, and with whom they are partnering. This could be useful to WHO and to programme managers in developing a coordinated approach.

Dr Leda Hernandez asked about the specific role of the LF programme manager in morbidity management, and considerable discussion focused on this. Dr Lisy Rasoazanamiarana reported that, in Madagascar, the role of the government has been to coordinate with NGDOs to set up the programme, provide training in morbidity management for health workers, and establish standards for patient follow-up. The NGDOs serve as an interface between the government and the community; their covenant with the community enables them to motivate community support for the programme and ensure adequate patient follow-up. Patients with ADL episodes are managed within the public health system. The Ministry of Health provides training on managing a variety of conditions of the lower limb, including diabetes and LF.

Dr Pepe agreed, and said that integration does not necessarily require combining the services of several NGDOs, all with separate disease mandates. Rather, a single interested NGDO with expertise in one area can, if motivated, help to address a variety of issues and diseases. Especially in low-income countries, flexibility is important.

Dr Brantus summarized the session and thanked the participants. He noted that the publication of WHO treatment guidelines will represent an important step forward. He called for developing clear strategies among interested parties, focused on integration based in partnership. He also acknowledged that NGDOs and Ministries of Health have different roles within a partnership, and clarity around these roles will facilitate explicit and specific action on the part of each partner. Integration of lower limb care is just one expression of the impulse toward greater integration of the health and medical systems now underway in many countries.

## Abbreviations

ADL: adenolymphangitis; ADB: African Development Bank; AFR: Africa Region of WHO; APOC: African Programme for Onchocerciasis Control. ComDT: community-directed treatment; DALY: disability-adjusted life year; DEC: diethylcarbamazine; DFID: UK Department for International Development; FY: fiscal year; GAELF: Global Alliance to Eliminate Lymphatic Filariasis; GPELF: Global Programme to Eliminate Lymphatic Filariasis; GSK: GlaxoSmithKline; IAD: Institute of Applied Dermatology; IDB: Inter-American Development Bank; ITN: insecticide-treated bednet; IU: implementation unit; JAF: Joint Action Forum of APOC; KCDC: Korea Centers for Disease Control and Prevention; LAC: Latin America and Caribbean; LF: lymphatic filariasis; MDA: mass drug administration; MDG: Millennium Development Goal; MDP: Mectizan Donation Program; mL: millilitre; MSD: Merck Sharpe & Dohme, Inc; MX: molecular xenodiagnosis; NGDO: non-governmental development organization; NTD: neglected tropical disease; PAHO: Pan American Health Organization; PCR: polymerase chain reaction; PCT: Preventive Chemotherapy and Transmission Control; PI: principal investigator; RAGFIL: rapid geographic assessment of bancroftian filariasis; RCG: Representative Contact Group of the GAELF; RFA: request for applications; RTI: Research Triangle Institute; SAE: serious adverse event; SEAR: Southeast Asia Region of WHO; STH: soil-transmitted helminth; USAID: US Agency for International Development; UN: United Nations; WHA: World Health Assembly; WHO: World Health Organization; WPR: Western Pacific Region of WHO.

## Competing interests

The authors declare that they have no competing interests.

## Authors' contributions

DA drafted the report.

## Supplementary Material

Additional file 1 **List of participants**. Click here for file
