# Supplementary material for: The 6th Meeting of the Global Alliance to Eliminate Lymphatic Filariasis: A half-time review of lymphatic filariasis elimination and its integration with the control of other neglected tropical diseases
Source: Parasit Vectors. 2010 Oct 20;3:100. doi: 10.1186/1756-3305-3-100 (PMC2984583; doi:10.1186/1756-3305-3-100)
Supplement: Additional file 1 — List of participants. [file 1756-3305-3-100-S1.DOC]

**Participants**

**Countries**

**Bangladesh**

Dr Israt Hafiz

Technical Consultant, Filariasis Elimination Program, Disease Control Unit, Directorate General of Health Services, Dhaka, 1206, Bangladesh

Email: [israthafiz@yahoo.com](mailto:israthafiz@yahoo.com); Tel: 88 01552 415070; Fax: 88 02 986 2994

**Burkina Faso**

Mr Windtare Roland Bougma

LF Program Manager, Ministry of Health, Direction de la Lutte Contre la Maladie, Programme National D’elimination de la Filariose Lymphatique, 03 BP 7009 Ouaga 03, Burkina Faso

Email: [wrolandbougma@yahoo.fr](mailto:wrolandbougma@yahoo.fr); Tel: 226 7027 0333; Fax 226 5031 5440

Mr Piga Prosper Tapsoba

Directeur de l'Administration et des Finances, Ministere de la Sante, 03 BP 7009 Ouaga 03, Burkina Faso

Email: [tapspiga@yahoo.fr](mailto:tapspiga@yahoo.fr); Tel: 00226 70200122; Fax: 00226 5032 6381

**Cameroon**

Dr Joseph Kamgno

Director, Filariasis Research Centre & Faculty of Medicine & Biomedical Sciences, Uinversity of Yaounde I, BP 5261, Yaounde

Email: [jkamgno@yahoo.fr](mailto:jkamgno@yahoo.fr); Tel: 237 7778 9736; Fax: 237 2220 2443

**CAR**Dr Louis Namboua

Director General de la Sante, Publique, BP 883, Bangul

Email : [namboua@yahoo.fr](mailto:namboua@yahoo.fr); Tel : 00 236 755 01834

**Cote D’Ivoire**

Dr Paul Maurice Dogbo Pepe

Schistosomiasis, STH and LF Programme Manager/MoH Republic of Ivory Coast, 06BP394, Abidjeu 06, Cote d’Ivoire

Tel: 00 225 23835

**Fiji**

Dr Eric Vilsoni Rafai

National Program Manager for Fiji Elimination of LF, Fiji Centre for Communicable Disease Control, Mataika House, Building 30, PO Box 16346, Suva, Fiji

Email: [eric.rafai@hotmail.com](mailto:eric.rafai@hotmail.com); Tel: 679 3320 066; Fax: 679 3320 344

**Ghana**

Dr Nana-Kwadwo Biritwum

Programme Manager, NTD Control Program, Ghana Health Service, PO Box MB-190, Accra, Ghana

Email: [nkadbiritwum@gmail.com](mailto:nkadbiritwum@gmail.com) or [nanakwadwo@ghsmail.com](mailto:nanakwadwo@ghsmail.com) ; Tel: 233 20 823 2286; Fax: 233 21 226 739

Dr Johnny Gyapong

Director, Research & Development Directorate, Ghana Health Service, P.O Box MB-190, Accra, Ghana

Email: [John.gyapong@ghsmail.org](mailto:John.gyapong@ghsmail.org); Tel: +233 24 426 5081; Fax: +233 21 685424

Dr Margaret Gyapong

Director, Dodowa Health Service Centre, Research and Development Directorate, Ghana Health Service, Box DD1, Ghana

Email: [Margaret.gyapong@ghsmail.org](mailto:Margaret.gyapong@ghsmail.org); Tel: 233 244 573138

**Haiti**
Dr Abdel Direny

NTD Country Program Manager, IMA World Health, 500 Main Street, P.O. Box 429, New Windsor MD, 21776, USA

Email: [abdeldireny@imaworldhealth.org](mailto:abdeldireny@imaworldhealth.org); Tel: 509 3816 5969

**India**

Professor Manokaran Gurusamy

Chief of Plastic Surgery and Lymphologist, Apollo Hospitals, 21 Greams Road, Chennai, 60006, India

Email: [manokaran_g@yahoo.com](mailto:manokaran_g@yahoo.com); Fax: 91 4428294429

Dr S Karthick

Director Gurumithrren Hopsital, Gurumithrren Hospital, 46 Chandragandhi Nagar, Ponmeni, Madurai 625-010, India
Email: [drkarthick@gurumithren.org](mailto:drkarthick@gurumithren.org); Web: www.gurumithrren.org

Professor Ranganatha Krishna Shenoy

Former Professor of Medicine, Filariasis Chemotherapy Unit, Near HDS Building, T.D. Medical College Hospital, Alappuzha, 688011, Kerala, India

Email: [drrkshenoy@gmail.com](mailto:drrkshenoy@gmail.com); Tel: 91477 2251 353

Dr S Sivasubramaniyan

Honorary Secretary Association of Surgeons of Rural India, Director Karthic Nursing Home, 47-A-Nayudu South Street, Settiarpatti-626122, Rajapalayamyia, Tamil Nadu, India

Email: [drsiva@gurumithrren.org](mailto:drsiva@gurumithrren.org)

Dr PK Srivastava

Joint Director National Vector Borne Disease Control Programme, Government of India, 22 Shamnath Mang, Delhi-110054, India

Mr Naveen Krishna Tarur

Program Manager, Institute of Applied Dermatology, Nayak’s Road, Kasaragod, Kerala, 67121 India

[residing address: 8-10/14 Balli Compound, B-2 Cross Kodikal, Mangalore, 575006, India]

Email: [naveenkrishnatarur@gmail.com](mailto:naveenkrishnatarur@gmail.com); Tel: 919 343248 744

**Indonesia**

Dr Rita Kusriastuti

Director of Vector Borne Disease Control, Ministry of Health Republic of Indonesia, Jln. Percetakan Negara No.29, Jakarta Pusat, Indonesia

Email: [ritakus@yahoo.com](mailto:ritakus@yahoo.com); Tel: 62 2142877586; Fax:62 21 4247573

**Ivory Coast**Mr Ekian Arsene Koua

General Management of the Budget, BP V57 Abidjan, Ivory Coast

Email: [arsene_koua@yahoo.fr](mailto:arsene_koua@yahoo.fr); Tel: 225 04181618

**Japan**

Professor Masamine Jimba

Department of Community and Global Health, The University of Tokyo, 7-3-1, Hongo, Bunkyo-Ku, Tokyo, 133-0033, Japan

Email: [mjimba@m.u-tokyo.ac.jp](mailto:mjimba@m.u-tokyo.ac.jp); Tel: 03 5841 3698; Fax: 03 5841 3422

Dr Shigeyuki Kano

Director, Department of Tropical Medicine and Malaria, Research Institute, National Center for Global Health and Medicine, 1-21-1 Toyama, Shinjuku, Tokyo, 162-8655, Japan

Email: [kano@ri.ncgm.go.jp](mailto:kano@ri.ncgm.go.jp); Tel: 813 3202 7181; Fax 813 3202 7364

Professor Eisaku Kimura

Professor, Department of Parasitology, Aichi Medical University, 21 Karimata, Yazako, Nagakute-cho, Aichi-Ken, 480-1195, Japan

Email: [kimura@aichi-med-u.ac.jp](mailto:kimura@aichi-med-u.ac.jp); Tel: 8156162 3311; Fax: 81 561 63 3645

Professor Kazuhiko Moji

Research Institute for Humanity and Nature, Motoyama 456-4, Kitaku Kamigamo, Kyoto, 603- 8047, Japan

Email: [moji-k@chikyu.ac.jp](mailto:moji-k@chikyu.ac.jp); Tel: 8175 707 2215; Fax: 8175 707 2509

Dr Hiroyoshi Endo

Professor, Tokyo Women’s Medical University, Department of International Affairs and Tropical Medicine, 8-1 Kawada-Cho, Shinjuku-Ku, 162-866, Japan

Email: [endo-hiroyoshi@w2.dion.ne.jp](mailto:endo-hiroyoshi@w2.dion.ne.jp); Tel: 813 5269 7421

**Kenya**

Ms Doris Wairimu Njomo

Research Scientist, Kenya Medical Research institute, 54840, 00200, Kenya

Email: [dnjomo@kemri.org](mailto:dnjomo@kemri.org); Tel: 254 020 2722541; Fax: 254 0202 720030

**Korea**

KiWoong Bae

Korea Association of Health Promotion

Jong-Yil Chai

Seoul National University. Email: cjy@snu.ac.kr

Hyeng Il Cheun

Ministry of Health and Welfare, Korea Centers for Disease Control and Prevention

FuHong Dai

Chungang University

Keeseon Eom

Chungbuk National University. Email: kseom@chungbuk.ac.kr

Jin HoKin

DaeWoo Hong

Shinpoong Pharm Co Ltd

SungTae Hong

Seoul National University

Sun Huh, Hallym University

JyeongKyu Jeon

Chungbuk National University

Hyangmin Cheong

NIH KCDC

Key Lee

Seuong Won Kang

NVRQS, Bacteriology & Parasitology Division

KiSang Kim

KNIH

Kisoon Kim

NIH KCDC

TaeIm Kim

Chungang University

TongSoo Kim

Department of Parasitology, Inha University Incheon 422-701, Korea

Email: tongsookim@inha.ac.kr; Tel: 82 32 890 0981

Youjin Kim

NIH KCDC

Jong Koo-Lee

Director/Deputy Minister, Ministry of Health and Welfare, Korea Centers for Disease Control and Prevention

Email: [docmohw@mohw.go.kr](mailto:docmohw@mohw.go.kr); Tel: 82 2 380 2600; Fax: 82 2 388 4601

Wam-Ja Lee

Director, Division of Malaria and Parasitic Diseases, Center for Immunology and Pathology, 194 Tongillo, Eunpyung, Seoul, 122-701 Korea

Email: [wonja@nih.go.kr](mailto:wonja@nih.go.kr); Tel: 822 380 2180

Joo-Shil Lee
Director General, Center for Immunology and Pathology, 194 Tongillo, Eunpyung, Seoul, 122-701 Korea

Email: [jooshil@nih.go.kr](mailto:jooshil@nih.go.kr); Tel: 82 2380 2150; Fax: 82 2382 6542

ChaeSeung Lim, Korea University College of Hedic

JhangHo Pak, University of Ulsan College of Hedic

JaeWon Park, Gachun University of Medicine

JaeYoung Park, Korea Association of Health Promotion

Hoogn Jeoung, Korea Association of Health Promotion

YunKyu Park, InHa University, School of Medicine

HyunOuk Song, KyungHee University

DongMin Lee, Chungbuk National University

HanSol Park, Chungbuk National University

Hyun Park, Wonkwang University

Han-Jon Rim

MyeongHeon Shin, Yonsei University College of Medicine

WoonMok Sohn, Gyeongsand National University

Professor Tong-Soo Kim

Inha University, Incheon, 400-712, Korea

Email: [tongsookim@inha.ac.kr](mailto:tongsookim@inha.ac.kr); Tel: 8232 8900981; Fax: 8232 8900982

TaiSoon Yong, Yonsei University College of Medicine

**Liberia**

Mr Chea Wesseh

Assistant Minister for Statistics/Ministry of Health, PO Box 10-909, 1000 Monrovia, 10 Liberia

Email: [cswesseh@yahoo.com](mailto:cswesseh@yahoo.com); Tel: 231 653 8603

Dr Louise Kpoto

Director of Epidemiology, Ministry of Health and Social welfare, 9009, Liberia

Email: [lkpoto@gmail.com](mailto:lkpoto@gmail.com), Tel: 231 77702609 or 231 77702609

**Madagascar**

Dr Lisy Rasoazanamiarana

LF Programme Manager, Ministry of Health, Programme Filariose BP 460 Antananarivo, II a 130 Bis Nanisana Antananarivo, 101, Madagascar

Email: [lisynirinarasoa@yahoo.fr](mailto:lisynirinarasoa@yahoo.fr)

**Malawi**

Mr Square Zakariya Mkwanda

LF Programme Manager, Ministry of Health, Box 30377, Lilongwe, Central Africa, Malawi

Email: [smkwanda@yahoo.com](mailto:smkwanda@yahoo.com); Tel: 265 8888 54425

Dr Majorie Chaponda

Ministry of health Advisor, Ministry of Health, Box 30377, Lilongwe, Central Africa, Malawi

Email: [chirwagln2006@yahoo.co.uk](mailto:chirwagln2006@yahoo.co.uk); Tel: 2651 789400

**Malaysia**

Professor Kok Hai Ong

Director of External Affairs, International Medical University, No 126, Jalan 19/155B, Bukit Jalil, Kuala Lumpur, 57000, Malaysia

Email: [kokhai_ong@imu.edu.my](mailto:kokhai_ong@imu.edu.my); Tel: 6032 731 7429; Fax: 6038 656 7232

**Mali**

Dr Benoit Dembele

Medical Doctor, FMPOS, MRTC, Filariasis Unit, Point G, Bamako 1805, Mali

Email: [benoit@icermali.org](mailto:benoit@icermali.org); Tel: 223 761 49193; Fax: 223 202 24987

**Mozambique**

Dr Olga Maria da Conceicăo Nelson Amiel

Program Manager to Eliminate LF in Mozambique, Ministry of Health, Eduardo Mondlane/Salvador Allend Av. Nu. 1008 Maputo, PO Box 264 Mozambique.

Email: [oamiel@misau.gov.mz](mailto:oamiel@misau.gov.mz); Tel: 258 82 7395150; Fax: 258 21 326 164

Dr Ricardo Thompson

Head of Department of Blood Parastiology, National Institute of Health, Av Eduardo Mondlane, 1008 Maputo 264, Mozambique

Email: rthompsonz@gmail.com; Tel: 25882 3060036; Fax: 258 21 431103

**Nepal**

Dr Garib Das Thakur

Director, Epidemiology and Disease Control Division, Department of Health Services, Ministry of Health and Population, Kathmandu, Nepal

Email: [thakurgd@gmail.com](mailto:thakurgd@gmail.com); Tel: 00977 14255796; Fax: 00977 1 4262268

Mr Kapildev Prasad Pajiyar

Advisor of Honourable Minister of Health and Population, Ministry of health and Population, Ramshah Path, Kathmandu, Nepal

Email: [kapildevpajiyar@gmail.com](mailto:kapildevpajiyar@gmail.com); Tel: 00977 1 4262534; Fax: 00977 14262565

**Papua New Guinea**

Dr Leo Sora Makita

Health Advisor Malaria and Vector Born Disease, National Department of Health, P.O. Box 807, Waigani, NCD Papua New Guinea

Email: [makitals@global.net.pg](mailto:makitals@global.net.pg); Tel: 675 301 3774; Fax: 675 301 3760

**Philippines**

Dr Leda Hernandez

Division Chief, Department of Health, Building 14, 3^rd^ Floor, San Lqazaro compound, Sta. Cruz, Manila, Philippines

Email: [dr_ledamher@yahoo.com](mailto:dr_ledamher@yahoo.com); Tel: 632 743 8301 LOCAL 2350 upto 2352; Fax: 632 711 7864

Dr Paulyn Jean Ubial

Assistant Secretary of Health, DOH Compound, Sta. Cruz, Manila, 1100, Philippines

Email: [paulyn_u@yahoo.com](mailto:paulyn_u@yahoo.com); Tel: 6327 116 180; Fax: 6327 116 180

**RD Congo**

Dr Gabin Mukiar Temor

Coorinator MTN

Email: [temormuk@yahoo.fr](mailto:temormuk@yahoo.fr)

**Senegal**

Professor Serigne Gueye

Professor and Chair of Urology, University Cheikh Anta DIOP, P.O Box 6039, Dakar, Senegal

Email: [smgueye@refer.sn](mailto:smgueye@refer.sn); Tel: 221 776 399 358

**Sri Lanka**

Dr Tilaka Savitri Liyange

Director, Ministry of Health, Central Leprosy Clinic, Room 21, National Hospital of Sri Lanka, Colombo 10, Sri Lanka

Email: [dashi@sltnet.lk](mailto:dashi@sltnet.lk); Tel: 94 011 271 4650

Dr Palitha Gunarathna Mahipala

Deputy Director General, Ministry of Healthcare and Nutrition, No 385 Nen, Baddegama Wimalawansa Thero Mawatha, Colombo 10, 01000, Sri Lanka

Email: [ddgphs@gmail.com](mailto:ddgphs@gmail.com); Tel: 94 11 2674682; Fax: 94 11 269 0035

**Tanzania**

Dr Mwelecele Ntuli Malecela

Acting Director General, National Institute for medical research, P.O Box 9653, Dar Es Salaam, Tanzania

Email: [mwelenutli@gmail.com](mailto:mwelenutli@gmail.com) or mmalecela@nimr.or.tz; Tel : 255 713 337092

Dr Upendo Mwingira

National Coordinator for NTD’s, Ministry of Health, Samara Avenue, P.O Box 9083, Dar Es Salaam, Tanzania

Email: [umwingira@yahoo.com](mailto:umwingira@yahoo.com); Tel 255 713262865; Fax: 255 222 2121360

Sidney Yongola

Associate Professor of Surgery, Consultant Urologist – MUHRS

Email: [swkyongola@yahoo.com](mailto:swkyongola@yahoo.com); Tel: 255 784 644445

**Thailand**

Miss Narumon Chanwimalueng

Ph.D Student/ Lymphedema specialist in TTDT, Lymphedema Day Care Center, Faculty of Tropical Medicine, Mahidol University, 420/6 Rajvithii Rd, Bangkok, Thailand 10400

Email: [nujrama@gmail.com](mailto:nujrama@gmail.com); Tel: 02 354 8395 (office) 085 368 2555 (mobile)

Miss Sumat Loimek

Medical Science Technician, Department of Prevention and Control Center 12, Thailand

Email: [sumark23@yahoo.co.th](mailto:sumark23@yahoo.co.th)

Mrs Kadkaew Meepiarn

Public Health Technical Officer, Bureau of Vector Borne Diseases Control, DDC, MOPH, Thailand

Email: [kk_mepian@yahoo.co.th](mailto:kk_mepian@yahoo.co.th)

Miss Sunsanee Rojanapanus

Public Health Technical Officer, Department of Disease Control, MOPH, Thailand

Email: [srojanapanus@yahoo.com](mailto:srojanapanus@yahoo.com)

Mrs Weena Santabutr

Public Health officer, Bureau of Vector Borne Diseases, Dept of Disease control, Nonthaburi, 11000, Thailand

Email: [wesantap52@gmail.com](mailto:wesantap52@gmail.com)

Dr Wichai Satimai

Director of Bureau of Vector Borne Diseases Control, Bureau of Vector Borne Diseases Control, Ministry of Public Health, Thailand

Email: [wichaisatimai@yahoo.co.th](mailto:wichaisatimai@yahoo.co.th)

Miss Piyaporn Suebtrakul

Lymphedema Day Care Center, Faculty of Tropical Medicine, Mahidol University, 420/6 Rajvithii Rd, Bangkok, Thailand 10400

Email: [g4248045@yahoo.com](mailto:g4248045@yahoo.com)

Anupong Sujariyakul

Director of Regional 4, DDC, MOPH
Email: [anupongho@yahoo.com](mailto:anupongho@yahoo.com)

Dr Saravudh Suvannadabba

Senior Preventive Expert in Medicine, Department of Disease Control, Ministry of Public Health, Tiwanon Road, Nonthaburi, 11000, Thailand

Email: [weapon1950@yahoo.com](mailto:weapon1950@yahoo.com)

Tanaporn Toothong

**Togo**

Dr Ameyo N. Monique Dorkenoo

Togo LF program Manager (Coordinator), Programmae National d'Elimination d ela filariose Lymphatique Togo, Ministère de la santé, BP 336 Lomé-Togo

Email: [monicadork@yahoo.fr](mailto:monicadork@yahoo.fr); Tel: 00 228904 4417 or 00 228 2200783; Fax: 00 228 2200783 or 00 228 222 0799

**Yemen**

Dr Abdul Samid Al-Kubati

National Focal point for LF, MOPH, Taiz, City of Light , P.O Box 6593, Yemen

Email: [a-samidku@hotmail.com](mailto:a-samidku@hotmail.com); Tel: 00967 7779 26030; Fax: 00967 424 2308

**Private Sector**

**GlaxoSmithKline**

Ms Minne Iwamoto

Manager, Global Community Partnerships, LF Programme, GlaxoSmithKline, One Franklin Plaza, FP 2130, 200N 16^th^ Street, Philadelphia, PA 19102, USA

Email: [minne.h.iwamoto@gsk.com](mailto:minne.h.iwamoto@gsk.com); Tel: 1 215 751 7096; Fax: 1 215 751 4046

Mr Andrew Wright

Director Disease Programmes, GlaxoSmithKline, 980 Great West Road, Brentford, Middlesex, TW8 9GS

Email: [andy.l.wright@gsk.com](mailto:andy.l.wright@gsk.com); Tel: 44 208 047 5515; Fax: 44 208 047 0684

**International Development Agencies and Donors**

**Bill and Melinda Gates Foundation**

Dr Jan Agosti

Strategic Program Team Lead, Senior Program Officer, Bill and Melinda Gates Foundation, P.O. Box 23350, Seattle WA, 98102, USA

Email: [jan.agosti@gatesfoundation.org](mailto:jan.agosti@gatesfoundation.org); Tel: 206 709 3331; Fax: 206 709 3170

Dr Julie Jacobson

Senior Program Officer, Bill and Melinda Gates Foundation, P.O. Box 23350, Seattle WA, 98102, USA

Email: [julie.jacobson@gatesfoundation.org](mailto:julie.jacobson@gatesfoundation.org); Tel: 206 770 1672; Fax 206 709 3170

**African Development Bank**
Dr Tshinko B. Ilunga

Health Division Manager , African Development Bank, B.P.323, Tunis Belvedere, Tunis, 1002, Tunisia

Email: [t.ilunga@afdb.org](mailto:t.ilunga@afdb.org); Tel: 2167 1102117; Fax: 2167 1333025

**Global Network for Neglected Tropical Diseases**

Dr Patrick Lammie

Technical Director, Global Network for Neglected Tropical Diseases. 2000 Pennsylvania Ave. Suite 7100, Washington DC, 20006, USA

Email: [pjl1@cdc.gov](mailto:pjl1@cdc.gov); Tel: 770 488 4054; Fax: 770 488 4253

**JICA**

Ms Kana Fukuhara

JICA Volunteer, JICA, Uday Tower, 7^th^ floor, 57 & 57A Glushan Avenue (South), Circle 1, Dhaka, 1212, Bangladesh

Email: [pon0728@livedoor.com](mailto:pon0728@livedoor.com); Tel: 880 1713 040276

Mr Tatsuya Misumi

JICA Volunteer, JICA, Uday Tower, 7^th^ floor, 57 & 57A Glushan Avenue (South), Circle 1, Dhaka, 1212, Bangladesh

Email: [icream11@yahoo.co.jp](mailto:icream11@yahoo.co.jp); Tel: 880 1713 040644

Mr Yasuhiro Miyaguni

JICA Volunteer, JICA, Uday Tower, 7^th^ floor, 57 & 57A Glushan Avenue (South), Circle 1, Dhaka, 1212, Bangladesh

Email: yasu.m.0516@gmail.com; Tel: 880 1713 040610

Miss Mika Yoshizawa

JICA Volunteer, JICA, Uday Tower, 7^th^ floor, 57 & 57A Glushan Avenue (South), Circle 1, Dhaka, 1212, Bangladesh

Email: [mikaringon-ringon@hotmail.co.jp](mailto:mikaringon-ringon@hotmail.co.jp); Tel: 8801713 040637

**RTI International**

Mary Linehan

Director of NTD control Program, RTI international, 805 15^th^ street NW, Washington DC, 20005 USA

Email: [melinehan@rti.org](mailto:melinehan@rti.org); [Tel:202](tel:202) 728 1964

Ms Katie Zoerhoff

M&E Associate, RTI International, 805 15^th^ Street NW, Washington DC 20005, USA

Email: [kzoerhoff@rti.org](mailto:kzoerhoff@rti.org); Tel: 202 974 7866; Fax: 202 974 7892

**RTI International - Nepal**

Dr Dharmpal Prasad Raman

Director, RTI/NTD Control Program – Nepal, Oasis Complex, Patan Dhooka, Lalitpur, 8975 Nepal

Email: [dpraman@np-ntd.rti.org](mailto:dpraman@np-ntd.rti.org); Tel: 977 1 5535780; Fax: 977 1 4266184

**USAID**

Angela Weaver

Neglected Tropical Disease Advisor, USAID, GH/HIDN/ID 3.07-27, Third Floor, RRB,1200 Pennsylvania Ave, NW, Washington DC 20523, USA

Email: [aweaver@usaid.gov](mailto:aweaver@usaid.gov); Tel: 1202 712 5603

**International Non-Governmental Organisations (NGO’s)**

**Carter Center**

Dr Frank O Richards

Director Lymphatic Filariasis Control, The Carter Center, 453 Freedome Parkway, One Copenhill Avenue, 30307, USA

Email: [frich01@emory.edu](mailto:frich01@emory.edu); Tel: 1 404 4203898; Fax: 1 404 4203 881

**Handicap International**

Dr Pierre Brantus

Consultant, Handicap International, 2 Rue du Reculet, Saint Genis Pouilly, 01630, France

Email: [brantus.pierre@orange.fr](mailto:brantus.pierre@orange.fr); Tel: 33 450 422 051; Fax 33 450 422452

**Helen Keller International**

Mr Chad MacArthur

Director- NTD Control, Helen Keller International, 352 Park Avenue South, 12^th^ floor, New York, NY 10010

Email: [cmacarthur@hki.org](mailto:cmacarthur@hki.org); Tel: 1 207 833 7344

**IMA World Health**

Dr Sarla Chand

Vice President, International Programs, IMA World Health, 500 Main Street, P.O. Box 429, New Windsor MD, 21776, USA

Email: [sarlachand@imaworldhealth.org](mailto:sarlachand@imaworldhealth.org) ; Tel: 410 635 8720

Ms Ann Varghese

Program Officer, IMA World Health, 500 Main Street, P.O. Box 429, New Windsor MD, 21776, USA

Email: [annvarghese@imaworldhealth.org](mailto:annvarghese@imaworldhealth.org); Tel: 410 635 8720

**LEPRA**

Mr Jose Randy de la Cruz

Senior Programs Officer, LEPRA Health in Action, 28 Middleborough, Colchester, CO1 2DG, UK

Email: [jose@leprahealthinaction.org](mailto:jose@leprahealthinaction.org); Tel: 0044 1206 216754

**Mectizan Donation Program**

Dr Adrian Hopkins

Director, Mectizan Donation Program, 325 Swanton Way, Decatur, 30030, USA

Email: [ahopkins@taskforce.org](mailto:ahopkins@taskforce.org); Tel: 1404 805 7719; Fax: 1 404 371 1138

Dr Yao K Sodahlon

Associate Director, LFE Section, Mectizan Donation Program, 325 Swanton Way, Decatur Ga, 30030, USA

Email: [ysodahlon@taskforce.org](mailto:ysodahlon@taskforce.org); Tel: 1404 388 6172; Fax: 1404 371 1138

**Sightsavers International**

Mr Simon Bush

Director African Alliances and Advocacy, Sightsavers, 21 NII Nortei Ababio Street, Airport. Accra, P.O. Box Kia 18190, Ghana

Email: [sbush@sightsavers.org](mailto:sbush@sightsavers.org); Tel: 233 21 774210; Fax: 233 21 780227

**World Vision**

Dr John Marfoh

Program Focal Person, NTD, World Vision Ghana, PMB Accra, Ghana
Email: [john_marfoh@wvi.org](mailto:john_marfoh@wvi.org); Tel: 233 24 435 8900, 233 24 4210764

**Global and Regional Programme Review Group Chairman**

**African Regional Office**

Dr Charles Ravaonjanahary

Chair, African Regional Programme Review Group, II A 130 Bis Nanisana Antananarivo, 101, Madagascar

Email: [ravac@moov.mg](mailto:ravac@moov.mg); Tel: 261 3207 59807; Fax: 261 2022 49287

**Americas Regional Office**

Dr Jean-Francois Vely

National Program Coordinator for Malaria Control, Ministry of Health and Population (Haiti), IMA World Health, 500 Main Street, P.O. Box 429, New Windsor MD, 21776, USA

Email: [joeve_44@yahoo.fr](mailto:joeve_44@yahoo.fr); Tel: 3404 4997/ 3755 5915

**Eastern Mediterranean Regional Office**

Professor Maged El-Setouhy

Professor of Public Health and Epidemiology, Chairman of the EMRO regional PRG for LF Elimination Programme, Ain Shams University, Department of Public Health, Abbasia, Cairo, Egypt

Email: [maged.elsetouhy@gmail.com](mailto:maged.elsetouhy@gmail.com); Tel: 2010 1783 248; Fax: 2022 261 3624

**PACELF**

Professor C. P. Ramachandran

8A-4~4 Belvedere, 1/63 off Jalan Tunku, Bukit Tunku, 50480 Kuala Lumpur, Malaysia

Email: [ramacp@hotmail.com](mailto:ramacp@hotmail.com); Tel: 00 603 2698 7275; Fax: 00 603 2698 6152

**South-East Asia Regional Office**

Dr Sombat Chayabejara

SEARO RPRG Chairman, 107 Pattanakan 53, Suanlang, Bangkok 10250, Thailand

Email: [sombatdr@yahoo.com](mailto:sombatdr@yahoo.com); Tel: 662 321 6442

**Academic and Research Institutes**

**Bonn University**

Professor Achim Hoerauf

Professor and Head, Institute of Medical Microbiology, Immunology and Parasitology, University Bonn Medical Center, Sigmund Freud St 25, Bonn 53105, Germany

Email: [hoerauf@microbiology-bonn.de](mailto:hoerauf@microbiology-bonn.de); Tel: 49 228 287 15675

Dr Sabine Susanne Mand

Institute of Medical Microbiology, Immunology and Parasitology (IMMIP), University Bonn Medical Center, Sigmund Freud St 25, Bonn 53105, Germany

Email: [mand@microbiology-bonn.de](mailto:mand@microbiology-bonn.de); Tel: 49 171 2653020; Fax: 49 228287 19573

**Georgetown University**

Professor Bernhard Liese

Chair, International Health Department, Georgetown University, 3700 Reservoir Road, NW, Washington DC 20057, USA

Email: [bhl6@georgetown.edu](mailto:bhl6@georgetown.edu); Tel: 1 202 687 3254; Fax: 1 202 784 3128

**James Cook University**Dr Alan Hauquitz

Senior Lecturer, James Cook University, Anton Breinl Centre for Public Health, Townsville, Queensland, 4811, Australia

Email: [alan.hauquitz@jcu.edu.au](mailto:alan.hauquitz@jcu.edu.au); Tel: 617 47816106; Fax: 61 74781 5254

**Liverpool School of Tropical Medicine**

Miss Lisa Bluett

IT & Communications Coordinator, Liverpool School of Tropical Medicine, Centre for Neglected Tropical Diseases, Pembroke Place, Liverpool, L3 5QA, UK

Email: [ljb@liv.ac.uk](mailto:ljb@liv.ac.uk); Tel: 0151 705 3242

Professor Moses Bockarie

Director of CNTD, Liverpool School of Tropical Medicine, Centre for Neglected Tropical Diseases, Pembroke Place, Liverpool, L3 5QA, UK

Email: [mjb12@liverpool.ac.uk](mailto:mjb12@liverpool.ac.uk); Tel: 0151 705 3343

Miss Rinki Deb

Research Assistant, Liverpool School of Tropical Medicine, Centre for Neglected Tropical Diseases, Pembroke Place, Liverpool, L3 5QA, UK

Email: [rinkideb@liv.ac.uk](mailto:rinkideb@liv.ac.uk); Tel: 0151 705 3131

Mrs Joan Fahy

Programme Manager, Liverpool School of Tropical Medicine, Centre for Neglected Tropical Diseases, Pembroke Place, Liverpool, L3 5QA, UK

Email: [fahy@liv.ac.uk](mailto:fahy@liv.ac.uk); Tel: 0151 705 3145

Miss Sara Holmes

Programme Administrator, Liverpool School of Tropical Medicine, Centre for Neglected Tropical Diseases, Pembroke Place, Liverpool, L3 5QA, UK

Email: [sara.holmes@liv.ac.uk](mailto:sara.holmes@liv.ac.uk); Tel: 0151 705 3145

Dr Louise Kelly-Hope

Project Manager, Liverpool School of Tropical Medicine, Centre for Neglected Tropical Diseases, Pembroke Place, Liverpool, L3 5QA, UK

Email: [lkhope@liv.ac.uk](mailto:lkhope@liv.ac.uk); Tel: 0151 705 3336

Professor David Molyneux

Senior Professorial Fellow, Liverpool School of Tropical Medicine, Centre for Neglected Tropical Disease, Pembroke Place, Liverpool, L3 5QA, UK

Email: [david.molyneux@liv.ac.uk](mailto:david.molyneux@liv.ac.uk); Tel: 0151 705 3291

Professor Mark Taylor

Professor of Parasitology, Liverpool School of Tropical Medicine, Head of Molecular and Biochemical Group, Pembroke Place, Liverpool, L3 5QA, UK

Email: [mark.taylor@liverpool.ac.uk](mailto:mark.taylor@liverpool.ac.uk); Tel: 0151 705 3112; Fax 0151 705 3771

**Lymphatic Filariasis Support Center, Atlanta**

Mr Brian Chu

Program Associate, TF for Global Health, 325 Swanton Way, Decatur, GA, 30030, USA

Email: [bchu@taskforce.org](mailto:bchu@taskforce.org); Tel: 1404 592 1427; Fax: 1404 371 1138

Dr Dominique Kyelem

Program Director, LF Support Center/Task Force for Global Health, 325 Swanton Way, Decatur, 30030, USA

Email: [dkyelem@taskforce.org](mailto:dkyelem@taskforce.org); Tel: 1404 687 5621; Fax: 1404 371 1138

Dr Eric Ottesen

Director LF support Center, Technical Director NTD control Program - RTI International, Task Force for Global Health, 325 Swanton Way, Decatur, GA 30030, USA

Email: [eottesen@taskforce.org](mailto:eottesen@taskforce.org); Tel: 1 404 6875604

Mr Alex Pavluck

Research Information Analyst, Mectizan Donation Program, 325 Swanton Way, Decatur, 30030, USA

Email: [apavluck@taskforce.org](mailto:apavluck@taskforce.org); Tel: 1 404 592 1421; Fax: 1 404 371 1138

**Pittsburgh University**

Dr Mary-Jo Geyer

Assistant Professor, University of Pittsburgh, Bakery Square, Suite 401, 6425 Penn Avenue, Pittsburgh Pennsylvania, 15206, USA

Email: [mjgeyer@pitt.edu](mailto:mjgeyer@pitt.edu); Tel: 412 624 6202; Fax: 412 624 6501

**Michigan State University**

Professor Charles Mackenzie

Professor, Pathobiology & Diagnostic Investigation, Michigan State University, A54 VMC, East Lansing, MI 48824, USA

Email: [mackenz8@msu.edu](mailto:mackenz8@msu.edu); Tel: 1 517 432 3644 Fax: 1 517 432 5836

**Notre Dame University**

Mr Logan Anderson

Financial Manager, University of Notre Dame, 1 Galvin Life Sciences, 46556, USA

Email: [landers7@nd.edu](mailto:landers7@nd.edu); Tel: 5746319705; Fax: 574 631 7413

**Smith College**

Dr Sandra Laney

Research Scientist, Smith College, Ford Hall, 100 Green Street, Northampton, MA 01085, USA

Email: [slaney@smith.edu](mailto:slaney@smith.edu); Tel: 413 585 4029

**Washington University**

Professor Gary Weil

Washington University School of Medicine, Infectious Diseases Division, Campus Box 8051, 660 South Euclid Ave, St Louis, MO 63110, USA

Email: [gweil@dom.wustl.edu](mailto:gweil@dom.wustl.edu); Tel: 1 314 454 7782; Fax: 1 314 454 5293

**World Health Organization**

**WHO Headquarters**

Dr Dirk Engels

Coordinator, Preventive Chemotherapy & Transmission Control, Avenue Appia 20, Geneva 27, CH 1211, Switzerland

Email: [engelsd@who.int](mailto:engelsd@who.int); Tel: 41 22 791 2726 (sec) 41 22 791 3824 (direct line); Fax: 41 22 791 4777

Dr Kazuyo Ichimori

Elimination of Lymphatic Filariasis, WHO, Avenue Appia 20, Geneva 27, CH 1211, Switzerland

Email: [ichimorik@who.int](mailto:ichimorik@who.int); Tel: 41227912767; Fax: 4122 791 4869

**WHO SEARO**

Professor Aditya Prasad Dash

Regional Advisor (Vector Borne & Neglected Tropical Diseases Control), South-East Asia Region, World Health Organisation, World Health House, Mahatma Gandhi Road, New Delhi 110 002, India

Email: [dasha@searo.who.int](mailto:dasha@searo.who.int); Tel: 91 995 899 4668; Fax: 91 11 2337 0197

**WHO WPRO**

Dr John Patrick Ehrenberg

Regional Advisor in Malaria and other vector borne Diseases, WHO, Western Pacific Regional Office, United Nations Avenue, cor Tuft Avenue, Manila, 1000, Philippines

Email: [ehrenbergj@wpro.who.int](mailto:ehrenbergj@wpro.who.int); [Tel: 632](tel:632) 528 9725; Fax: 632 521 1036

Dr Ah Sian Tee

Director, Combating Communicable Diseases, WHO, Western Pacific Regional Office, United Nations Avenue, cor Traft Avenue, Manila, 1000, Philippines

Email: [teea@wpro.who.int](mailto:teea@wpro.who.int); Tel: 632 528 9701; Fax: 632 521 1036

**Rapporteur**

Dr David Addiss

Senior Program Officer, Fetzer Institute, 9292 West KL Ave, Kalamazoo, MI, 49009, USA

Email: [dgaddiss@yahoo.com](mailto:dgaddiss@yahoo.com); Tel: 1269 760 0360; Fax: 1 269 372 2163
